# Biochemical and molecular profiling of induced high yielding M_3_ mutant lines of two *Trigonella* species: Insights into improved yield potential

**DOI:** 10.1371/journal.pone.0305691

**Published:** 2024-07-29

**Authors:** Neha Naaz, Sana Choudhary, Nazarul Hasan, Nidhi Sharma, Nora M. Al Aboud, Wael F. Shehata

**Affiliations:** 1 Department of Botany, Cytogenetics and Plant Breeding Lab, Aligarh Muslim University, Aligarh, India; 2 Department of Biology, Faculty of Science, Umm Al-Qura University, Makkah, Saudi Arabia; 3 Department of Agricultural Biotechnology, College of Agriculture and Food Sciences, King Faisal University, Al-Ahsa, Saudi Arabia; Dr Rammanohar Lohia Avadh University, INDIA

## Abstract

*Trigonella*, commonly known as Fenugreek, is among the most promising medicinal herbs consumed worldwide due its protein rich dietary contributions. This study involved induced mutagenesis on two *Trigonella* species (*Trigonella foenum-graecum* var. PEB and *Trigonella corniculata* var. Pusa kasuri) using caffeine and sodium azide as mutagens, resulting in the identification of nine high-yielding mutant lines in the M_3_ generation. Molecular characterization using SCoT markers revealed a high polymorphism of 28.3% and 46.7% in PEB and Pusa kasuri, respectively, facilitating the investigation of genetic divergence among the control and mutant lines. Similarity correlation analysis indicated a high similarity between mutant A and mutant C (0.97) and between mutant J and mutant O (0.88), while the lowest similarity was observed between mutant B and mutant F (0.74) and between control and mutant L (0.58). Mutant F and Mutant J displayed the highest seed yield and its attributing traits, and seed protein content in PEB and Pusa kasuri, respectively. Physiological parameters, including chlorophyll content (Mutants A and N) and carotenoids (mutant A and J), exhibited improvements. Assessment of stomatal and seed characteristics using scanning electron microscopy may lead to improved physiological processes and distinction at the interspecific level, respectively. Methanolic extracts of the control and the mutant lines of both species were subjected to GC-MS analysis, revealing 24 major phytocompounds known for their pharmacological activities (antioxidant, anti-inflammatory, anticancer, etc.). Statistical methods such as Pearson correlation heatmap and pairwise scatter plot matrix provided insights into the correlations and linear associations among parameters for both PEB and Pusa kasuri. The strong correlation between iron content and seeds per pod in the mutant lines suggests a promising avenue for further research. Continued research and breeding efforts using these mutants can lead to significant advancements in agriculture and medicine, benefiting farmers, consumers, and industries alike.

## Introduction

The world’s population is projected to increase from 7.2 billion to 9.6 billion by 2050, with further growth expected to reach 10.9 billion by the year 2100. This rapid increase raises concerns about the balance between food supply and demand [1]. In response to this challenge, the Food and Agriculture Organization (FAO) of the United Nations acknowledges the necessity of providing adequate and affordable food through sustainable agricultural practices, as outlined in their report "The Future of Food and Agriculture: Trends and Challenges" [2]. The adoption of superior smart farming techniques has gained traction due to the need to address environmental challenges and climate change impacts while ensuring the production of high-quality, nutritious food [3]. Among the various food crops, leguminous plants rank as the second most significant crop, contributing 27% to global primary food production. These plants are crucial due to their ability to fix atmospheric nitrogen, serving as a valuable protein source and playing a pivotal role in agriculture [4].

Nevertheless, modern factors including lifestyle choices, dietary habits, stress, environmental influences, and the extensive utilization of synthetic chemicals in food processing and agriculture have worsened the occurrence of numerous human diseases. Documentation of natural remedies obtained from plants spans several millennia. Integrating medicinal herbs into diets is a well-known strategy for preventing a range of diet-related illnesses like diabetes, cancer, inflammation, and cardiovascular diseases [5].

Fenugreek, an ancient herb, has been utilized in Central Asia since 4000 BC [6]. This annual leguminous herb, predominantly cultivated in India and accounting for 80% of global production. Additionally, fenugreek serves multiple purposes, its leaves are utilized as a vegetable, dried leaves as an herb, and seeds as a spice. The root, seed, and stalk of fenugreek contain significant metabolites such as trigonelline, diosgenin, neotycogenin, and yamogenin, which are highly valued in the steroid industries [7]. Fenugreek seeds consist of about 58% carbohydrates, 23–26% proteins, 0.9% lipids, and 25% fiber, whereas the leaves contain approximately 6% carbohydrates, 4.4% proteins, and 1.1% fiber [8]. Both fenugreek leaves and seeds are widely utilized to prepare extracts and powders for traditional use in therapeutic applications treating various health ailments such as eczema, burns, gout, diarrhoea, abdominal distension, and stomach discomfort, among others. They are rich in essential minerals and vitamins such as calcium, potassium, iron, magnesium, phosphorus, β carotene, copper, zinc, and vitamin C [9]. Fenugreek seeds also contain flavonoids, volatile and fixed oils, with high levels of linoleic acid, linolenic acid, and oleic acid in the oils. Therefore, substantial pharmacological and clinical evidence has emphasized the medicinal uses of fenugreek seeds, including their potential for anti-diabetic, hypolipidemic, anti-obesity, anticancer, anti-inflammatory, antioxidant, antifungal, antibacterial activities, and more. These benefits are attributed to its bioactive components. The extraction and categorization of these bioactive compounds have resulted in the development of specialized medicines with potent activity profiles [10]. Gas chromatography-mass spectrometry (GC-MS) has recently been adopted to identify various bioactive constituents within medicinal plants [11]. Recently, scanning electron microscopy (SEM) has become an invaluable implement in various biological research fields, offering high-resolution imaging to study ultrastructure features not possible with Light Microscopy [12].

Assessing the genetic diversity of germplasm is crucial for identifying genotypes with higher diversity and better performance in specific conditions. The absence of comprehensive data regarding genetic diversity, inter- and intra-specific variability, and genetic relationships among *Trigonella* species hampers large-scale production and the development of enhanced varieties. Gene characterization and evaluation of genetic variation offer valuable insights to plant breeders, these findings present opportunities to develop superior varieties featuring traits preferred by breeders. However, various methods exist for evaluating genetic diversity, with molecular markers being preferred due to their advantages over other techniques. DNA markers are detectable in all plant tissues, irrespective of the growth stage or developmental status of the plants. Molecular markers offer valuable insights into genetic diversity and relationships among various crop species, particularly in the realm of crop breeding. Start Codon Targeted (SCoT) polymorphisms serve as reproducible markers derived from the short-conserved region surrounding the ATG translation start codon in plant genes. SCoT markers offer an efficiency advantage over arbitrary markers because of their longer primer distances and high annealing temperatures [13, 14]. SCoT markers have been used in various plant species, including *Trigonella*, wheat, grape, and sugarcane, for tasks like evaluating genetic polymorphism, identifying cultivars, mapping QTLs, and performing DNA fingerprinting [15–18]. Several studies have demonstrated the ability of SCoT markers to distinguish genotypes associated with specific traits such as disease resistance and abiotic stress tolerance [19, 20]. Furthermore, SCoT markers exhibit higher stability, repeatability, and reliability in contrast to RAPD, ISSR, and AFLP markers, making them well-suited for genetic mapping across various plants and for marker-assisted selection programs [21].

The absence of characterization and assessment research may restrict the selection, improvement, and expansion of cultivation. As a result, the current study bears particular importance in the continuous endeavors to expedite fenugreek enhancement and establish core collections capable of efficiently supporting traditional breeding initiatives.

Therefore, the work aimed to

- study the yield attributing traits, physiological parameters, seed protein and mineral contents in the induced high yielding M_3_ mutants of both the varieties (PEB and Pusa kasuri).
- identify the bioactive compounds in the methanolic extracts of the high yielding mutant seeds through gas chromatography-mass spectrometry (GC-MS).
- study the variations in the stomatal morphology and seed micromorphology in high yielding mutants.
- study the genetic variability between the induced mutants by utilizing SCoT markers.

## Materials and methods

### Plant material

Certified and healthy seeds of *Trigonella foenum-graecum* (var. PEB) and *Trigonella corniculata* (var. Pusa kasuri) were acquired from the Indian Agricultural Research Institute (IARI), New Delhi, India. The description of both species is illustrated in Supplementary file (S1 Table in S1 File).

### Mutagen doses, mutagen treatments and selection of mutants

In the present experiment, *Trigonella* seeds were treated with caffeine and sodium azide (manufactured by Sigma Aldrich, Mumbai, India) to induce mutagenesis. Initially, the seeds were soaked in double distilled water (DDW) for 24 hours, followed by exposure to varying doses of caffeine and sodium azide (0.2%, 0.4%, 0.6%, 0.8%, and 1.0%) at room temperature for 9 hours. Treated and untreated seeds were sown in 5 replications (30 seeds each) in an earthen pots during the rabi season (mid-October 2019) to raise the M_1_ generation. Regular watering and weeding were carried out during the cultivation process.

### M_2_ generation

The seeds harvested from M_1_ plants were collected separately from both the treated and untreated populations. Five healthy randomly selected seeds were sown, for inducing M_2_ generation during mid-October 2021. During the M_2_ generation, treated plants underwent screening, resulting in the identification of 30 mutant lines with high yields. These lines were selected based on stable traits such as plant height (cm), branches per plant, pods per plant, seeds per pod, seed weight (g), and overall plant seed yield (g). These selected mutant lines were then carried forward for further study in the M_3_ generation.

### M_3_ generation

All seeds from the M_2_ generation were harvested individually, and 20 healthy seeds from high-yielding mutant lines were planted to initiate the M_3_ generation in mid-October 2022. Nine high-yielding mutants (5 from *Trigonella foenum graecum* denoted as Mutant A, Mutant B, Mutant C, Mutant F, Mutant G and 4 from *Trigonella corniculata* denoted as Mutant J, Mutant L, Mutant N, Mutant O) were screened based on plant seed yield (Table 1). The controls (C1 and C2) and these mutants underwent evaluations, including yield and physiological parameters, seed mineral contents, Scanning Electron Microscopy, GC-MS analysis, and molecular characterization to develop their relative profiles.

**Table 1:** High yielding M_3_ mutant lines isolated from the mutagenic treatments in *Trigonella foenum graecum* (PEB) and *Trigonella corniculata* (Pusa kasuri).

S.No.	Mutant codes	Species	Mutagen used	Concentrations	Characters
*Trigonella foenum-graecum* (PEB)					
**1**	C1	PEB	-	-	Normal height with average yield
**2**	A	PEB	Caffeine	0.2%	Tall mutant with increased yield
**3**	B	PEB	Caffeine	0.4%	Semi dwarf mutant with moderately increased yield
**4**	C	PEB	Caffeine	0.6%	Tall mutant with high yield
**5**	F	PEB	SA	0.2%	Tall mutant with high yield
**6**	G	PEB	SA	0.4%	Semi dwarf mutant with increased yield
*Trigonella corniculata* (Pusa kasuri)					
**7**	C2	Pusa kasuri	-	-	Normal height with average yield
**8**	J	Pusa kasuri	Caffeine	0.2%	Tall mutant with increased yield
**9**	L	Pusa kasuri	Caffeine	0.6%	Normal height mutant with moderately increased yield
**10**	N	Pusa kasuri	SA	0.2%	Tall mutant with high yield
**11**	O	Pusa kasuri	SA	0.4%	Semi dwarf mutant with increased yield

### Yield parameters

The mutant lines were evaluated for various yield parameters, including plant height (cm), number of branches per plant, number of pods per plant, number of seeds per pod, and yield per plant (g).

### Chlorophyll and carotenoid content

The pigment content of leaves was determined using the [22] method. Fresh leaves (1g) were ground to a fine pulp in 20 ml of 80% acetone using a mortar and pestle. Following homogenization, the mixture underwent centrifugation at 5000 rpm for 10 minutes, and the resulting supernatant was transferred into a 100 ml volumetric flask. The volume was calibrated by the addition of 80% acetone. Absorption measurements for chlorophyll were recorded at wavelengths of 645 and 663 nm, whereas for carotenoids, readings were taken at 480 and 510 nm using a spectrophotometer. Pigment content was determined using the equation specified in reference [23].

$$Total\;chlorophyll\;content\;(g^{-1}\;fresh\;leaves)=[20.2\;(OD645)+8.02\;(OD663)]V/(1000\times w)$$ (1)

$$Carotenoid\;content\;(g^{-1}\;fresh\;leaves)=[7.6\;(OD480)\;-1.49\;(OD510)]V/(1000\times w\times d)$$ (2)

Wherever,

V, the volume of an extract; W, the mass of leaf tissues; d, the length of light path (1.4cm).

### Proline content

To assess the proline content of leaves, the [24] method was used. 0.5 grams of fresh leaves were homogenized in 10 milliliters of a 3% aqueous solution of sulphosalicylic acid. The homogenized mixture was subsequently collected and centrifuged at 6000 rotations per minute (rpm) for 15 minutes. A 2 mL portion was withdrawn and combined with 2 mL of glacial acetic acid and ninhydrin. The mixture was then heated in a water bath for 1 hour. The reaction was halted by transferring the test tube into an ice bath and extracting it with 4 mL of toluene, followed by stirring for 20–30 seconds. The chromatophore in toluene was extracted, and the intensity of the red color was assessed at 520 nm. The total proline content was determined using a standard curve prepared with standard proline.


μMolespergtissue=μgproline/ml×mltoluene/115.5×5/gsample (3)

### Assessment of protein and mineral element in the seeds

The protein content of the seeds in the mutants was determined using the "Lowry Assay: protein by Folin reagent" [25]. The determination of mineral elements in the seeds (Iron—Fe and Copper—Cu) was conducted via an atomic absorption spectrophotometer (AAS) following Gupta’s method [26]. Ground seed samples (0.5g) from both mutants and control groups were weighed and transferred into a 50 ml digestion tube. About 5 ml of an acid mixture (1 sulphuric acid, 4 perchloric acid, and 10 nitric acid) were added into each sample via Pyrex funnel, and the tube was placed into the block digestor. Initially, the samples were heated at 60°C for 15 minutes until the reaction ceased, as confirmed by the eruption of fumes. The samples were subsequently heated to 120°C until they became colorless. Afterwards, the tubes were removed from the digestor and allowed to cool. The blank was also prepared without a seed sample of only an acid mixture solution. The digested samples and blanks were diluted with 50 ml of double distilled water (DDW), and mineral elements (Fe, Zn, and Cu) were analyzed using Atomic Absorption Spectroscopy (AAS) with standard solutions for calibration. The wavelengths of the lamp were maintained at 372.0 nm for Fe, 307.6 nm for Zn, and 327.4 nm for Cu during the evaluation process. The total mineral contents were directly estimated using AVANTA 2.0 software preinstalled in the AAS computer equipment.

### Examination of stomatal behavior in leaves and the micromorphology of seeds using scanning electron microscopy (SEM)

Scanning electron microscopy was utilized to examine both stomatal aperture and seed micromorphology. The same protocol was executed as outlined by [27]. This analysis was accomplished with a SEM(JEOL), JSM-6510LV, JAPAN microscope at 15kV at various magnifications. The size of the stomata was measured in μm. The SEM micrographs were acquired at the USIF, Aligarh Muslim University, Aligarh.

### Preparation of methanolic extract

In the study, 5g of seeds from both the control and mutants were powdered using a mortar and pestle. The powdered seeds were then soaked in 10 ml of methanol for 24 hours. Following this, the methanolic seed extract underwent centrifugation at 10,000 rpm for 15 minutes. The resulting pellet was then discarded, and the supernatant was filtered through Whatman filter paper No.1. Subsequently, the filtered extract was collected and preserved in vials for further analysis.

### GC-MS analysis of methanolic extract

The GC-MS analysis of the methanolic extract was conducted using a GC-MS-TQ8050 NX instrument (Shimadzu Corporation, Kyoto, Japan) at the Central Instrumentation Laboratory of the Central University of Punjab, Bathinda, India. The split injector was adjusted to 250°C with a split ratio of 5. Subsequently, the oven temperature was raised gradually from 0 to 40°C, followed by an increase to 220°C withhold times of three and five minutes, respectively. Following this, the temperature was elevated to 250°C and maintained for 5 minutes. Helium was employed as the carrier gas with a steady flow rate of 1 mL min^-1^. The ion source temperature was adjusted to 230°C, while the interface temperature was set at 250°C. The mass scan range encompassed 40–800 amu. The column flow rate was held constant at 1.00 mL min^-1^, with the column oven temperature set to 40°C. The sample loading and injection volume were both 1 μL. The analysis of bioactive compounds in the methanolic extract was conducted using the NIST17R library and NIST17M2 library, based on their m/z ratio and retention time.

### SCoT (Start Codon Targeted) marker analysis

SCoT marker analysis was carried out to investigate the genetic variations of the isolated mutants of *Trigonella foenum-graecum* and *Trigonella corniculata*. Genomic DNA extractions were performed on lyophilized leaves using a modified CTAB (Cetyltrimethylammonium bromide) based protocol [28].

#### Agarose gel electrophoresis

The appropriate amount of agarose was added to TBE buffer to prepare a 0.8% solution. The agarose was fully dissolved in the buffer by heating the mixture to 80–85˚C in a microwave oven and then cooled to 50˚C.

#### DNA Quantification

The qualitative and quantitative evaluation of total genomic DNA was conducted by subjecting the DNA samples to electrophoresis on 0.8% agarose gels [29].

#### SCoT marker amplification

The PCR reaction mixture of 10μl contained all the components required for PCR analysis. The standard PCR mixture for SCoT marker analysis comprised 50 ng of genomic DNA in 1x reaction buffer, 1.5 mM MgCl_2_, 10 pmol primer, and 1 unit of Taq DNA polymerase (BioTools) in a reaction volume of 10 μl. All PCR reactions were conducted using a Gene Amp PCR 9700 Thermal Cycler. The PCR was carried out in a thermocycler, with a total of 35 cycles for SCoT marker analysis. The SCoT markers profiles of samples were visualized on 1.5% agarose gel.

#### Data analysis

The amplified fragments (bands) produced were manually scored for their presence (indicated as ’1’) or absence (indicated as ’0’). The genetic similarity (GS) values between pairs of samples were calculated based on Jaccard’s similarity coefficient. The similarity matrix was employed to create a phenetic dendrogram using the UPGMA (Unweighted Pair Group Method of Arithmetic Averages) algorithm, as proposed by Sneath & Sokal [30], to group the samples into clusters based on their similarities. The statistical analysis was performed using NTSYSpc 2.21 software.

### Statistical analysis

The experiments were conducted with five replicates for each treatment, and data were presented as the mean ± standard error. Statistical analysis was conducted using R software, employing one-way ANOVA to ascertain the least significant difference (LSD) between treatments at significance levels of *p* < 0.05 (5%) and *p* < 0.01 (1%). Additionally, Pearson correlation heatmap and pairwise scatter plot matrix with linear regression lines were created using Python version 3.11.3 to explore relationships and associations between variables in the data.

## Results

### Yield parameters, physiological parameters, and proline content of high yielding mutant lines

Table 2 presented data on various yield parameters and physiological characteristics of high-yielding mutant lines alongside their corresponding controls in two plant species, *Trigonella foenum-graecum* and *Trigonella corniculata*. In *T*. *foenum-graecum*, mutant F exhibited the highest seed yield (3.96g), fertile branches (7.00), pods per plant (37.00), and seeds per pod (9.52) under 0.2% SA treatment. In *T*. *corniculata*, mutant J had the highest seed yield (8.32g), fertile branches (8.00), clusters per plant (37.00), and pods per cluster (40.00) under 0.2% caffeine treatment. Overall, several mutants (A, B, C, F, G, J, L, N, and O) of both species showed increased yield parameters compared to their controls.

**Table 2:** Mean values of yield attributing traits, physiological parameters, proline, protein and mineral content of M_3_ high yielding mutant lines.

Codes	Plant height (cm)	Fertile branches/plant	Clusters/plant	Pods/plant (or cluster)	Seeds/pod	Yield/plant (g)	Total chlorophyll (mg g^-1^)	Carotenoids (mg g^-1^)	Proline (μg g^-1^)	Protein (%)	Iron (mg g^-1^)	Copper (mg g^-1^)
*Trigonella foenum-graecum* (PEB)												
C_1_	82.22	3.00	-	17.00	8.00	1.45	2.69	0.43	24.38	19.20	4.15	3.29
**A**	93.53	6.00	-	35.00	8.42	3.62	3.21	1.09	25.90	24.25	5.23	3.55
**B**	62.51	4.00	-	24.00	8.53	1.85	3.12	0.98	30.15	20.54	5.20	2.10
**C**	89.52	6.00	-	34.00	8.46	3.12	2.94	0.65	34.27	23.15	4.55	1.95
**F**	92.25	7.00	-	37.00	9.52	3.96	3.09	1.05	26.05	26.47	6.37	2.67
**G**	73.45	5.00	-	42.00	8.53	2.92	3.02	0.95	31.53	21.62	5.18	2.62
*Trigonella corniculata* (Pusa kasuri)												
C_2_	79.25	4.00	27.00	21.00	7.00	6.50	2.23	0.41	25.33	18.12	3.94	2.93
**J**	88.13	8.00	37.00	40.00	7.88	8.32	2.94	0.93	26.00	20.25	4.05	2.99
**L**	75.37	5.00	29.00	29.00	7.33	7.27	2.25	0.52	30.95	19.88	3.93	2.73
**N**	87.55	7.00	36.00	34.00	8.45	8.15	3.06	0.85	25.88	19.32	4.26	3.10
**O**	53.65	8.00	36.00	31.00	8.12	8.02	2.54	0.76	27.10	17.56	4.10	3.02

The physiological parameters of the high-yielding mutants also showed improvements. Mutants A and N of PEB and Pusa kasuri, respectively, exhibited significant increases in chlorophyll content (3.21 mg g^-1^ and 3.06 mg g^-1^, respectively) related to the controls (Table 2). Similarly, mutant A of PEB and mutant J of Pusa kasuri had the highest carotenoid content (1.09 mg g^-1^ and 0.93 mg g^-1^, respectively). Proline content significantly increased in mutant C and mutant L (34.27 μg g^-1^ and 30.95 μg g^-1^, respectively).

In summary, the study demonstrated that specific mutants showed enhanced yield parameters and physiological characteristics, indicating their potential for improved crop performance.

### Protein and mineral element content in seeds of high-yielding mutant lines

In the M_3_ high-yielding mutant lines of PEB and Pusa kasuri, mutants A and F showed the best elevation in seed protein content. Mutants J and L also exhibited significant enhancements in protein content. Minor protein content improvements were observed in mutants B, C, G, N, and O compared to their respective controls. Mutant F of PEB had the highest protein content (26.47%) compared to its control (19.20%), and mutant J of Pusa kasuri showed the maximum increase (20.25%) compared to the control (18.12%). Regarding seed mineral elements, mutants F and N had the highest Fe content (6.37 mg g^-1^ and 4.26 mg g^-1^, respectively), while mutants A and N had the highest Cu content (3.55 mg g^-1^ and 3.10 mg g^-1^, respectively). Thus, specific high-yielding mutants exhibited notable improvements in protein content and mineral element contents in the seed, indicating their potential for crop improvement (Table 2).

### Examination of stomatal behavior of leaves and seed micromorphology of high-yielding mutant lines

**Fig 1:**
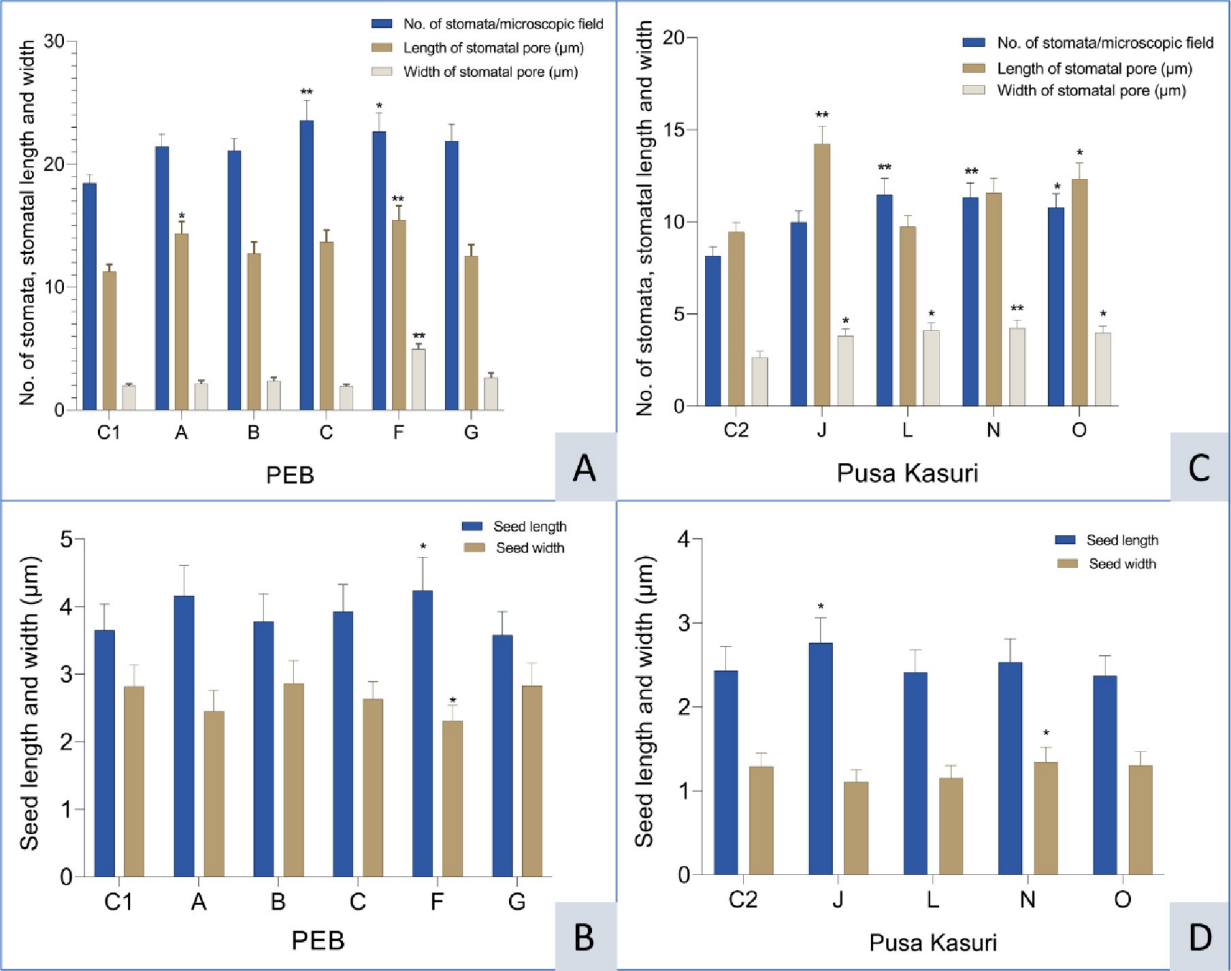
Number of stomata per microscopic field, length and width of stomatal pore and seed length and width of *Trigonella foenum-graecum* (A, B) and *Trigonella corniculata* (C, D). Bars are represented as mean ± SE. Values with (*) and (**) asterisk differ significantly at p< 0.05 and p< 0.01, respectively.

**Fig 2:**
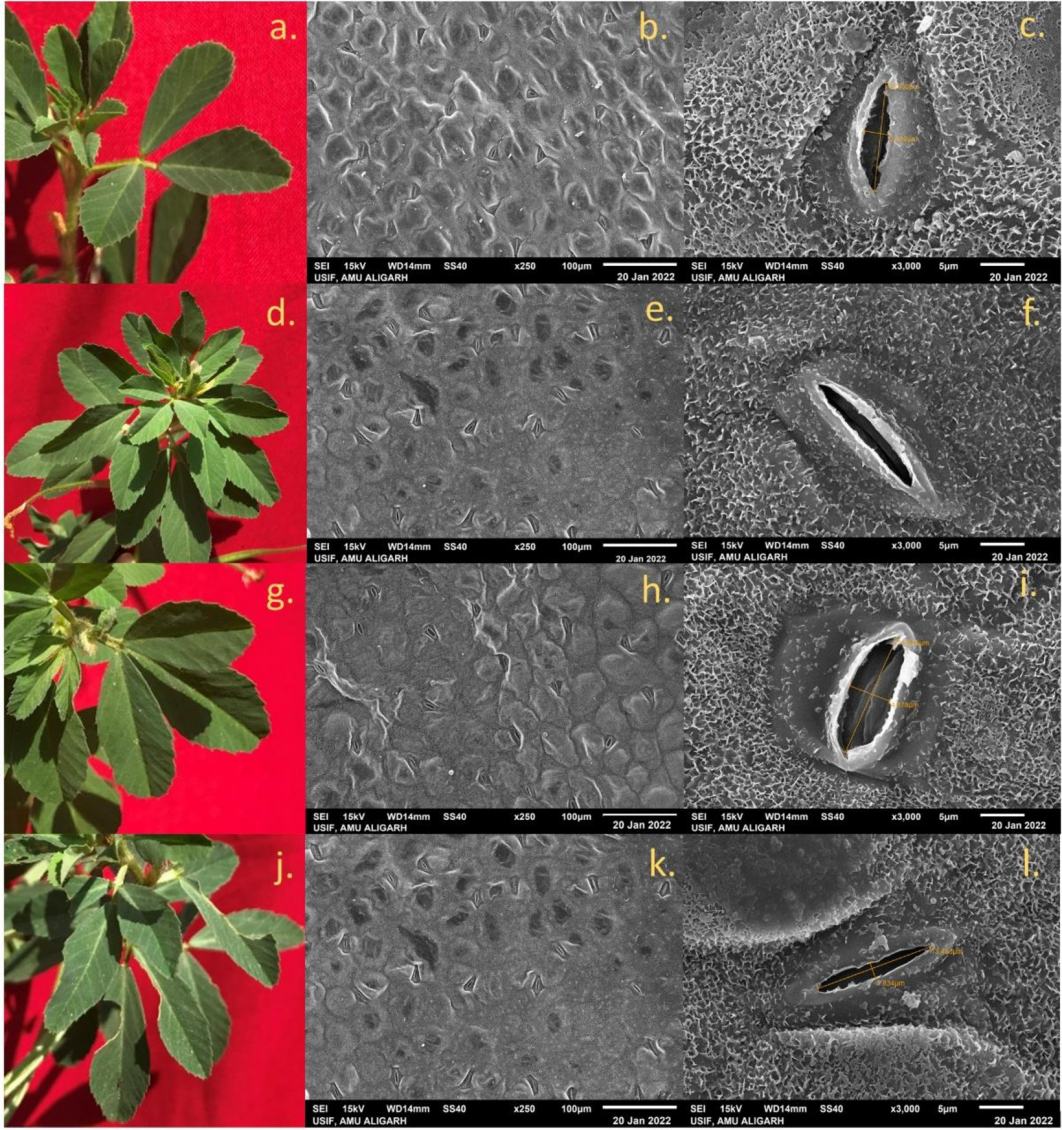
Morphological pictorial of leaf and SEM microphotographs showing number of stomata per microscopic field and dimensions of stomatal pore of control C1 (a, b, c) mutant A (d, e, f) mutant C (g, h, i) and mutant F (j, k, l) of *Trigonella foenum-graecum*.

**Fig 3:**
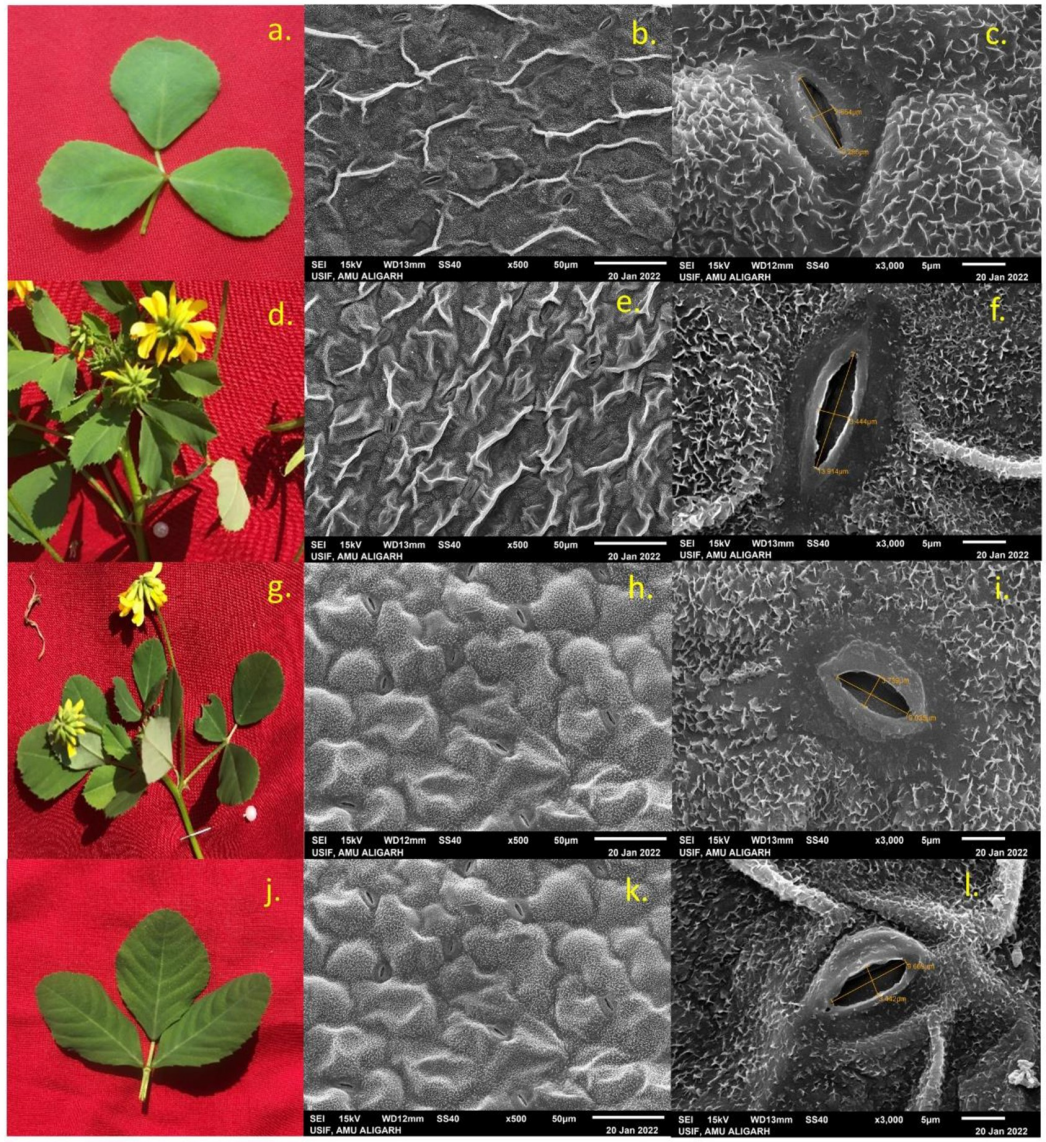
Morphological pictorial of leaf and SEM microphotographs showing number of stomata per microscopic field and dimensions of stomatal pore of control C2 (a,b,c) mutant J (d,e,f) mutant N (g,h,i), and mutant O (j,k,l) of *Trigonella corniculata*.

**Fig 4:**
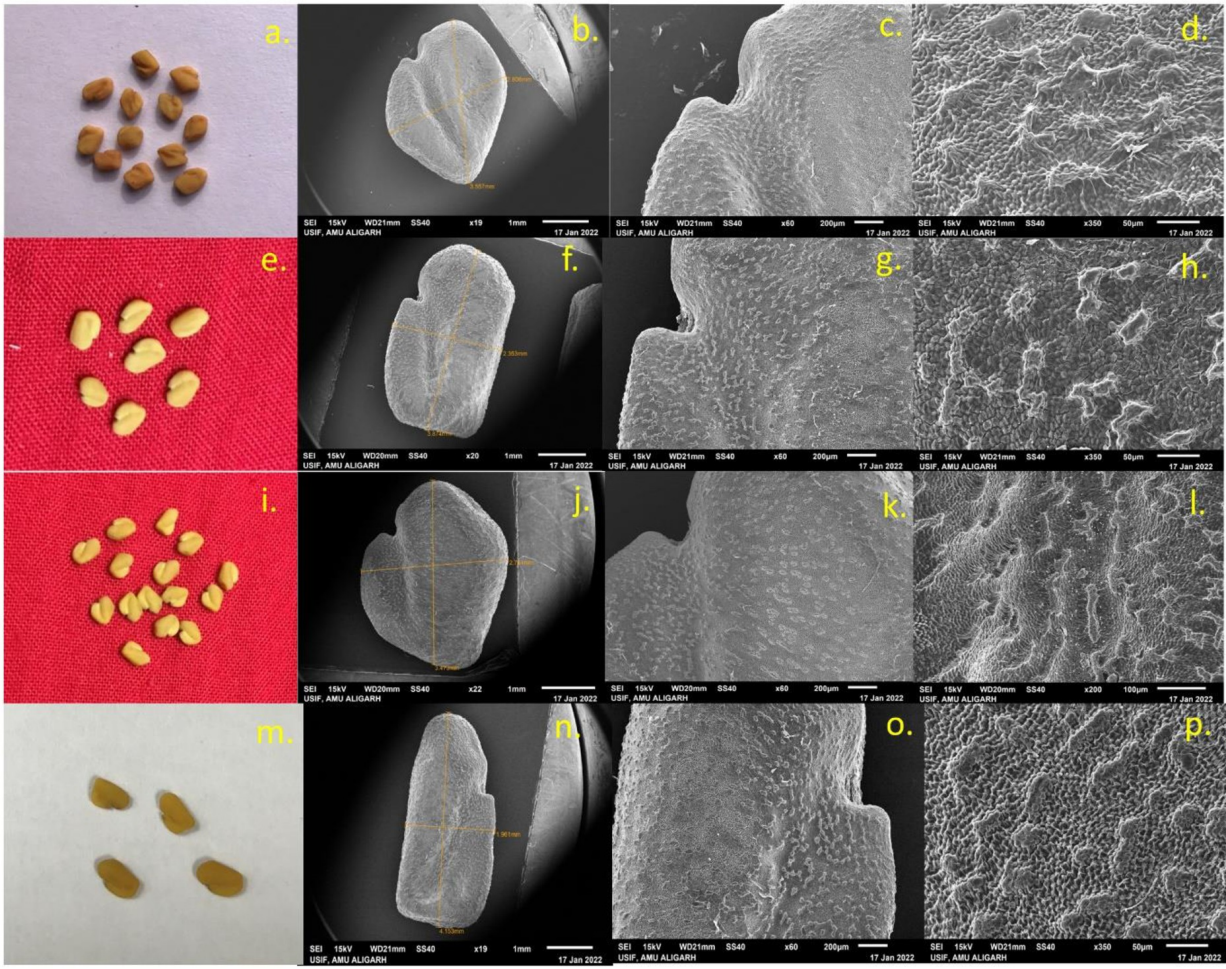
Morphological pictorial of seeds and SEM microphotographs showing seed morphology, hilum shapes and seed coat pattern of control C1 (a,b,c,d), mutant A (e,f,g,h), mutant C (i,j,k,l), mutant F (m,n,o,p) of *Trigonella foenum-graecum*.

**Fig 5:**
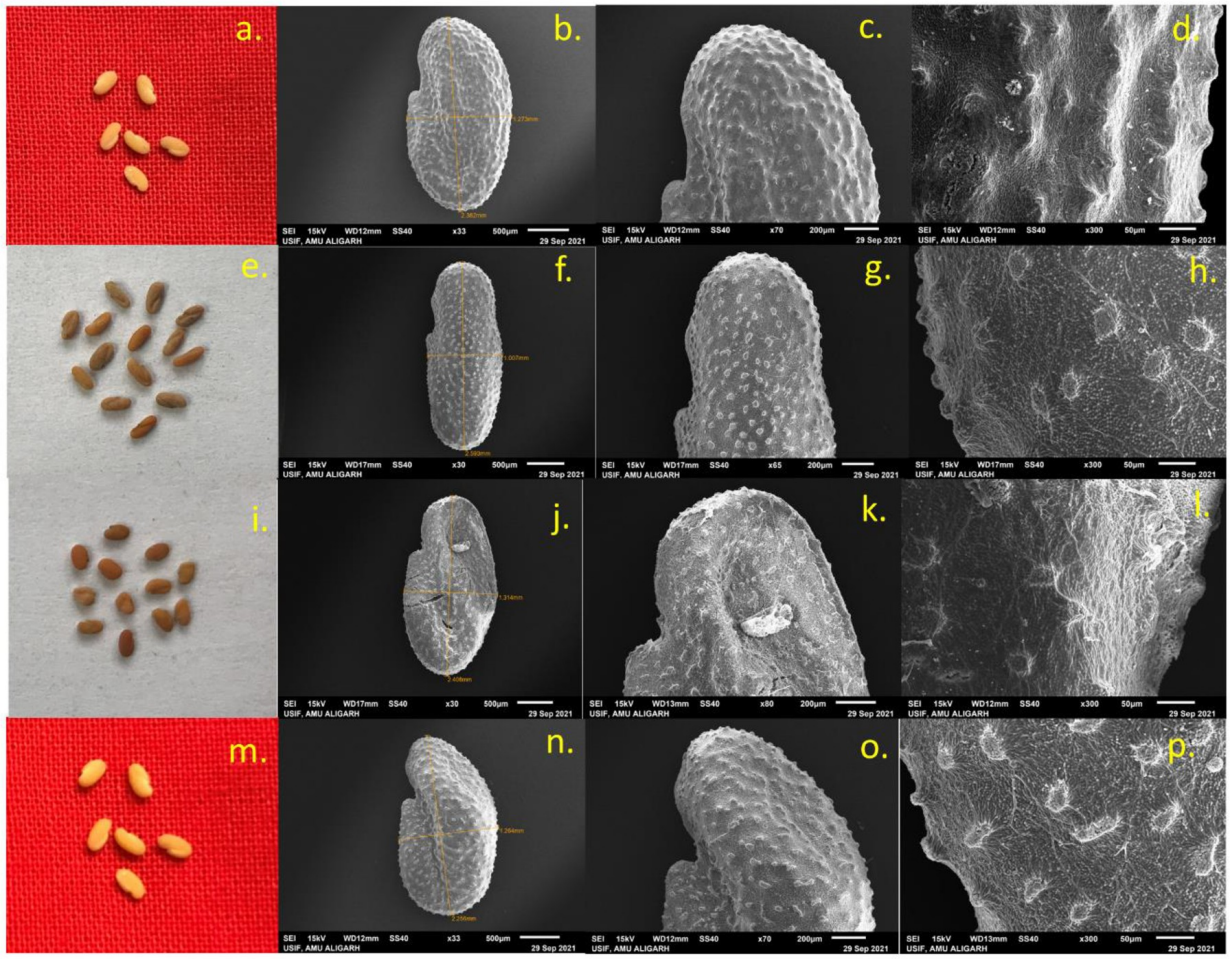
Morphological pictorial of seeds and SEM microphotographs showing seed morphology, hilum shapes and seed coat pattern of control C2 (a,b,c,d), mutant J (e,f,g,h), mutant N (i,j,k,l), and mutant O (m,n,o,p) of *Trigonella corniculata*.

Scanning electron microscopy of high-yielding mutant lines’ stomatal behavior and seed micromorphology was carried out, and the results were presented in Figs 1–5. In PEB, mutant C exhibited the maximum number of stomata per microscopic field (23.57), while in Pusa kasuri, mutants L showed the highest number of stomata per microscopic field (11.48) at a 1% significant level. Mutants A and F of PEB and mutants J and O of Pusa kasuri displayed a remarkable increase in stomatal length, while other mutants exhibited slight changes compared to the control. The most substantial increases in length of the stomata were noticed in mutant F and mutant J (15.45 μm and 14.23 μm) compared to the respective controls. Regarding the width of stomatal pores, mutants F and N (4.96 μm and 4.24 μm) exhibited the highest significant increase, both at a 1% level (p<0.01), compared to their controls (Fig 1A and 1C). The variations in the shape and size of stomatal pore of *T*. *foenum-graecum* and *T*. *corniculata* are displayed in Figs 2 and 3.

In this study, the external and internal surface of the seed, as well as seed shape, length, and width, were analyzed. The observations revealed variations in morphological characteristics such as seed shape, arrangement of surface cells, and size. Mutants of both species exhibit notable variations in length and width of the seed. Mutant F and mutant J had highest seed length (4.24 μm and 2.76 μm), while mutant G and mutant O had smallest seed length (3.58 μm and 2.37 μm) among all mutants. Mutant F and mutant N had highest seed width (2.31 μm and1.34 μm), while in mutant F and mutant J smallest seed width (2.31 μm and 1.11 μm) was noted (Fig 1B and 1D). Seed shape could also potentially play a significant role in identification. Various forms were seen such as rhomboid, rectangular, oblong, etc. in PEB (Fig 4) while elliptic, oblong, oval, and ovate shapes were noted in Pusa kasuri (Fig 5). The ultrastructural examination of the seed coat of the mutants of both species illustrates characteristic cell pattern. Seed coat patterns such as papillate and mounded with papillae, having isodiametric, tangentially elongated, irregular, and irregular reticulate seed coat cells outlines were noticed.

In summary, the high-yielding mutant lines showed variations in stomatal characteristics and seed micromorphology, with certain mutants exhibiting significant improvements related to their corresponding controls.

### Phytochemical investigation of methanolic seed extract of high yielding mutant lines by GC-MS

**Fig 6:**
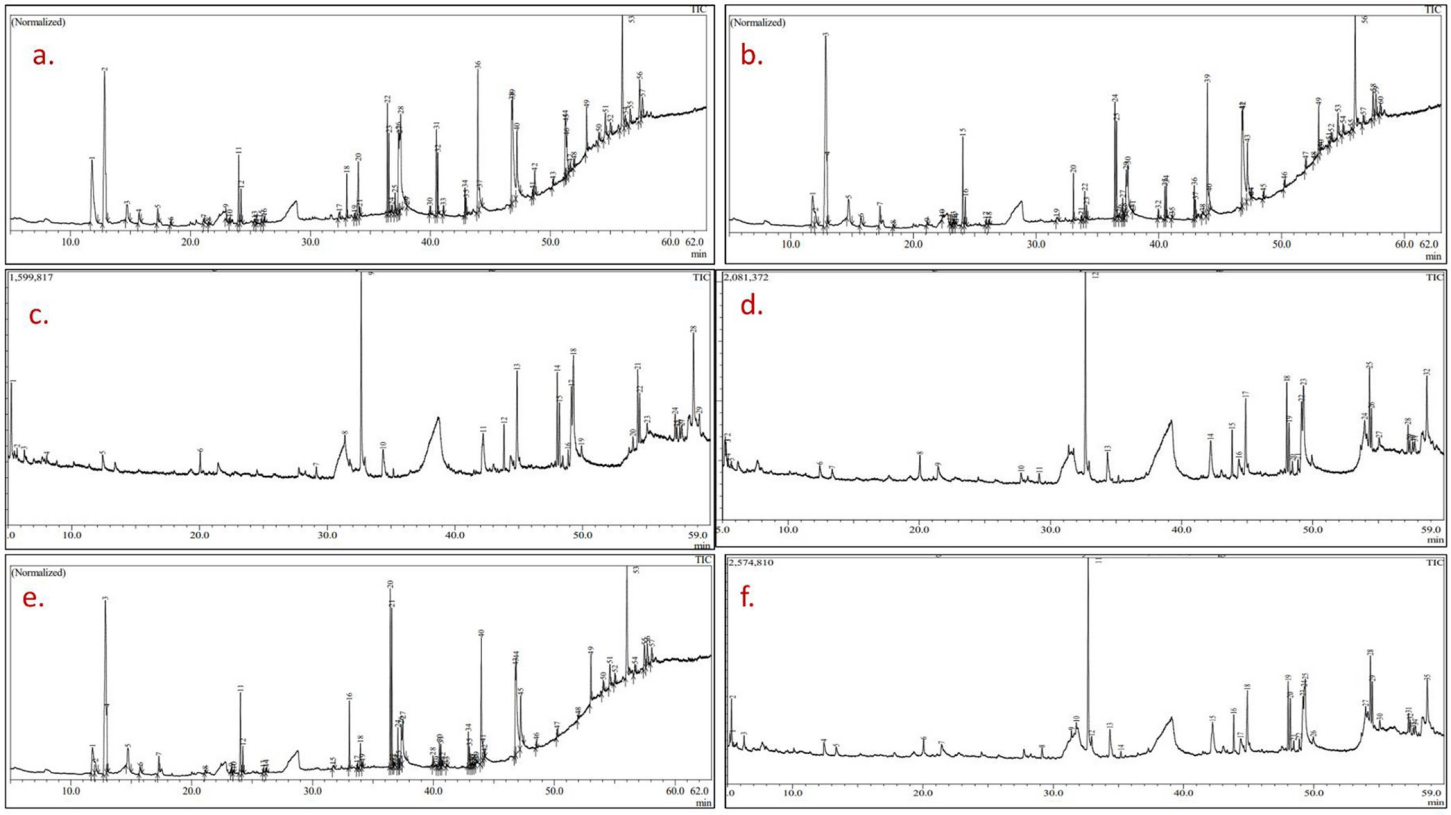
GC-MS chromatogram of the seed methanolic extract of control C_1_ (a), mutant A (b), mutant B (c), mutant C (d), mutant F (e) and mutant G (f) of *Trigonella foenum-graecum*.

**Fig 7:**
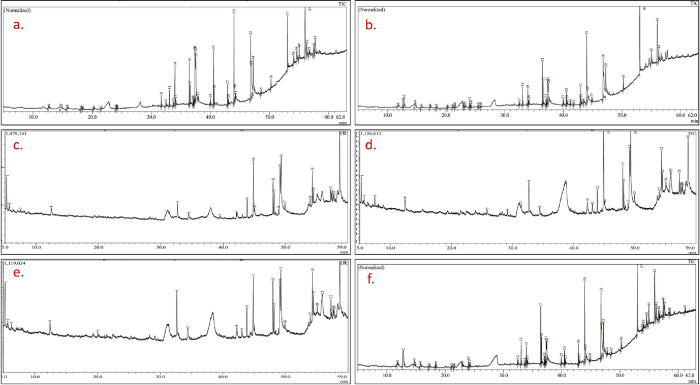
GC-MS chromatogram of the seed methanolic extract of control C_2_ (a), mutant J (b), mutant L (c), mutant N (d), and mutant O (e) of *Trigonella corniculata*.

The GC-MS chromatogram of the methanolic seed extract of the mutants of PEB and Pusa kasuri, presented in Figs 6 and 7, respectively, showed the presence of various bioactive phytochemicals. The methanolic extract contained significant phytochemical elements, including trihydroxy alcohol, ketones, thiophene, esters, fatty acids, amines, fatty amides, etc. These compounds were identified based on their retention time and peak area percentage, to those of known compounds listed in the National Institute of Standards and Technology (NIST) library (S2 and S3 Tables in S1 File). All the mutant lines exhibited variations in peak presence, compound composition, and area percentages. The composition of major phytocompounds found in the methanol extracts of both species is displayed in Table 3, along with their class, molecular formula and weight, compound ID, and pharmacological activities. Among the mutants isolated from PEB, mutant A had the maximum number of bioactive constituents, followed by mutants F, B, G, and C. In Pusa kasuri, the maximum bioactive compounds were detected in mutant J, followed by mutants L, O, and N. The major identified bioactive compounds in the methanolic extracts of PEB included cyclohexanamine, N-methyl; 1H-Azepin-1-amine, hexahydro-; δ-Dodecalactone; 2-Naphthalenol, decahydro-; Hexadecanoic acid, methyl ester; n-Hexadecanoic acid; 9,12-Octadecadienoic acid (Z, Z)-, meth; 9-Octadecenoic acid, methyl ester, (E)-; Octadecanamide, N-(2-hydroxyethyl)-; 9,12-Octadecadienoic acid (Z, Z)-; Hexadecanoic acid, 2-hydroxy-1-(hydro; and others. In Pusa.

**Table 3:** Major phytoconstituents identified from the methanolic extracts of the control and the mutant lines of *T*. *foenum graecum* and *T*. *corniculata*.

Phytocompounds	Class of compounds	Molecular formula	M.W. (g/mol)	Pubchem CID	IUPAC name	Activities	References
Glycerin	trihydroxyalcohol	C_3_H_8_O_3_	92.09	753	propane-1,2,3-triol	Antiherpetic and antiviral	[31]
2,4-Dihydroxy-2,5-dimethyl-3(2H)-furan	Ketone	C_6_H_8_O_4_	144.12	538757	2,4-dihydroxy-2,5-dimethylfuran-3-one	Antioxidant	[32]
2-Methoxythiophene	Thiophene	C_5_H_6_OS	114.17	85610	2-methoxythiophene	Antifungal	[33]
Cyclohexanamine, N-methyl-	Aliphatic amine	C_7_H_15_N	113.2	7514	N-methylcyclohexanamine	No activity reported	-
1H-Azepin-1-amine, hexahydro-	**-**	C_6_H_14_N_2_	114.19	22198	azepan-1-amine	No activity reported	**-**
.delta.-Dodecalactone	Ester	C_12_H_22_O_2_	198.3	12844	6-heptyloxan-2-one	No activity reported	-
2-Naphthalenol, decahydro-	-	C_10_H_18_O	154.25	13216	1,2,3,4,4a,5,6,7,8,8a-decahydronaphthalen-2-ol	Antineoplastic, anti-infectives and antioxidant	[34]
3-Nitro-4-hydroxypyridine	-	C_5_H_4_N_2_O_3_	140.1	228506	3-nitro-1H-pyridin-4-one	No activity reported	-
1H-Azepine, hexahydro-3,3,5-trimethyl-	-	C_9_H_19_N	141.25	118239	3,3,5-trimethylazepane	No activity reported	-
Hexadecanoic acid, methyl ester (Methyl palmitate)	Fatty acid esters	C_17_H_34_O_2_	270.5	8181	methyl hexadecanoate	Antimicrobial, antioxidant, hypocholesterolemic, pesticide, lubricant, antiandrogenic and hemolytic 5-alpha-reductase inhibitor	[35, 36]
n-Hexadecanoic acid (Palmitic acid)	Saturated fatty acid	C_16_H_32_O_2_	256.42	985	hexadecanoic acid	Antibacterial and antifungal	[37]
9,12-Octadecadienoic acid (Z,Z)-, methy (Linoleic Acid)	omega-6 fatty acid (unsaturated)	C_18_H_32_O_2_	280.4	5280450	(9Z,12Z)-octadeca-9,12-dienoic acid	Antioxidant, anticancer, antimicrobial, Anti-inflammatory, hypocholesterolemic, antiarthritic, anticoronary, antieczemic and antiacne.	[38–40]
9-Octadecenoic acid, methyl ester, (E)- (Methyl elaidate)	Fatty acid	C_19_H_36_O_2_	296.5	5280590	methyl (E)-octadec-9-enoate	Antioxidant, Antibacterial and antifungal	[41]
9,12,15-Octadecatrienoic acid, methyl e (Methyl elaidolinolenate)	Fatty acid	C_19_H_32_O_2_	292.5	5367462	methyl (9E,12E,15E)-octadeca-9,12,15-trienoate	antimicrobial	[42]
9,12-Octadecadienoyl chloride, (Z,Z)- (Linoleoyl Chloride)	Fatty acid	C_18_H_31_ClO	298.9	9817754	(9Z,12Z)-octadeca-9,12-dienoyl chloride	Antioxidant, antibacterial, anti-nociceptive, anti-inflammatory, larvicidal and repellent effects	[43–45]
Cis-9-hexadecenal	mono-unsaturated fatty-aldehyde	C_16_H_30_O	238.41	5364643	(Z)-hexadec-9-enal	Antioxidant, antifungal and antimelanogenic	[46, 47]
9-Tetradecenal, (Z)-	-	C_14_H_26_O	210.36	5364471	(Z)-tetradec-9-enal	No activity reported	-
Octadecanamide, N-(2-hydroxyethyl)-	Fatty amides	C_20_H_41_NO_2_	327.5	27902	N-(2-hydroxyethyl)octadecanamide	Anti inflammatory	[48]
Hexadecanoic acid, 2-hydroxy-1-(hydro (2-Palmitoylglycerol)	Lipids	C_19_H_38_O_4_	330.5	123409	1,3-dihydroxypropan-2-yl hexadecanoate	Antiinfective, Anti-inflammatory and Antiprotozoal	[40]
Vitamin E (Alpha-Tocopherol)	Vitamins	C_29_H_50_O_2_	430.7	14985	(2R)-2,5,7,8-tetramethyl-2-[(4R,8R)-4,8,12-trimethyltridecyl]-3,4-dihydrochromen-6-ol	Antioxidant, antiageing analgesic, anti-alzheimer, anti-dermatitic, anti-diabetic, anti-inflammatory, anti-osteoarthritic, anti-proliferant, antispasmodic, anti-sunburn, antitumor, hypocholesterolemic, immunostimulant	[49–51]
6,9-Octadecadienoic acid, methyl ester	Fatty acid ester	C_19_H_34_O_2_	294.5	5365662	methyl (6E,9E)-octadeca-6,9-dienoate	Antifungal	[52]
9-Octadecenoic acid (Z)-, 2,3-dihydroxy	Fatty ester	C_21_H_40_O_4_	361.6	71751310	(1,1,2,3,3-pentadeuterio-2,3-dihydroxypropyl) (Z)-octadec-9-enoate	Anti tumor	[53]
Stigmast-5-en-3-ol, oleate	Steroidal ester	C_47_H_82_O_2_	679.2	20831071		Anticancer	[54]
Carbamic acid, 2-(dimethylamino)ethyl	Amines	C_5_H_12_N_2_O_2_	132.16	48131	2-(dimethylamino)ethyl carbamate	No activity reported	-
Bis(2-(Dimethylamino)ethyl) ether	Amines	C_8_H_20_N_2_O	160.26	18204	2-[2-(dimethylamino)ethoxy]-N,N-dimethylethanamine	Anti tumor	[55]

kasuri, the major bioactive compounds were found: 2-Naphthalenol, decahydro-; Hexadecanoic acid, methyl ester; n-Hexadecanoic acid; 9,12-Octadecadienoic acid (Z,Z)-, methyl; 9,12-Octadecadienoyl chloride, (Z,Z)-; 9,12-Octadecadienoic acid (Z,Z)-; Octadecanamide, N-(2-hydroxyethyl)-; Hexadecanoic acid, 2-hydroxy-1-(hydro; and others. In summary, the GC-MS analysis indicated the presence of diverse bioactive compounds in both species, with significant differences among the mutant lines in terms of compound composition and abundance.

### SCoT marker analysis of high yielding mutants

Six primers were employed to investigate the genetic diversity among the mutants using SCoT molecular markers. All tested primers yielded reproducible and scorable bands in both species, as depicted in S1, S2 Figs in S1 File. showcasing the SCoT pattern. The SCoT primers produced 50 and 63 scorable bands in PEB and Pusa Kasuri, respectively, averaging 8.33 and 10.5 bands per primer. From this total number of bands, 14 (28.3%) and 31 (46.7%) were identified as polymorphic, with an average of 2.33 and 5.17 bands per primer in PEB and Pusa kasuri, respectively. In PEB, the highest number of polymorphic bands (4) was produced by SCoT3 and SCoT12, while the lowest number of polymorphic bands (1) was observed with SCoT7, SCoT26, and SCoT27. The highest polymorphism (50%) was observed with SCoT3, while the Polymorphic Information Content (PIC) values ranged significantly from 0.24 in SCoT3 to 0.03 in SCoT27 in PEB. Whereas in Pusa kasuri, the highest polymorphism (67%) was produced from SCoT3 and SCoT17, and PIC values generated from all primers varied from 0.25 in SCoT17 to 0.10 in SCoT12. Notably, the SCoT3 primer displayed the highest polymorphism percentage in both species, making it an effective marker to distinguish control and mutant populations (Table 4).

**Table 4:** List of SCoT primers, their GC content, total bands generated and polymorphic information of each primer.

Sr No.	Primer	Sequences (5’-3’)	%G/C	Tm	*T*. *foenum graecum*					*T*. *corniculata*				
					Total band	Monomorphic band	Polymorphic band	% polymorphism	PIC value	Total band	Monomorphic band	Polymorphic band	% polymorphism	PIC value
**1**	SCoT3	CAACAATGGCTACCACCG	56	50°C	8	4	4	50%	0.24	15	5	10	67%	0.21
**2**	SCoT12	ACGACATGGCGACCAACG	61	50°C	10	6	4	40%	0.13	10	7	3	30%	0.10
**3**	SCoT17	ACCATGGCTACCACCGAG	67	50°C	9	8	1	11%	0.03	12	4	8	67%	0.25
**4**	SCoT19	ACCATGGCTACCACCGGC	67	50°C	7	4	3	43%	0.17	8	4	4	50%	0.18
**5**	SCoT26	ACCATGGCTACCACCGTC	61	50°C	8	7	1	13%	0.03	9	6	3	33%	0.12
**6**	SCoT27	ACCATGGCTACCACCGTG	61	50°C	8	7	1	13%	0.03	9	6	3	33%	0.14
**Total**					**50**	**36**	**14**	**170**		**63**	**32**	**31**	**280**	
**Average**					**8.33**		**2.33**	**Av. = 28.3%**	**0.11**	**10.5**		**5.17**	**Av. = 46.7%**	**0.17**

Based on Jaccard’s coefficient, the genetic similarities indicated that the highest value was observed between mutant A and mutant C, with a value of 0.97, while the lowest similarity value was found between mutant B and mutant F, with a value of 0.74 in PEB. On the other hand, the highest similarity coefficient was 0.88 among mutant J and mutant O, while the minimal similarity coefficient was 0.58 among the control and mutant L in Pusa kasuri (S4 and S5 Tables in S1 File).

Phylogenetic trees were constructed for the mutants based on the Unweighted Pair Group Method of the Arithmetic Averages (UPGMA) analysis of SCoT data. In the SCoT marker system, the dendrogram of PEB exhibited a division into two primary clusters, with the first cluster including both the control and mutant B. The second cluster grouped the remaining four mutants. The second cluster was further subdivided into two subclusters, with the first subcluster consisting of three mutants (mutant A, mutant C, and mutant G), while the second subcluster have only mutant F. Meanwhile, the dendrogram of Pusa kasuri featured one major cluster, which grouped four mutants, along with another cluster solely including the control (the most divergent among all). The major cluster was further subdivided into two subclusters. The first subcluster exclusively contained mutant L, while the second subcluster was subsequently divided into two groups. The first group included two mutants (mutant J and mutant O), while the second group involved only mutant N (S3 Fig in S1 File).

### Pearson correlation heatmap and pairwise scatter plot matrix

**Fig 8:**
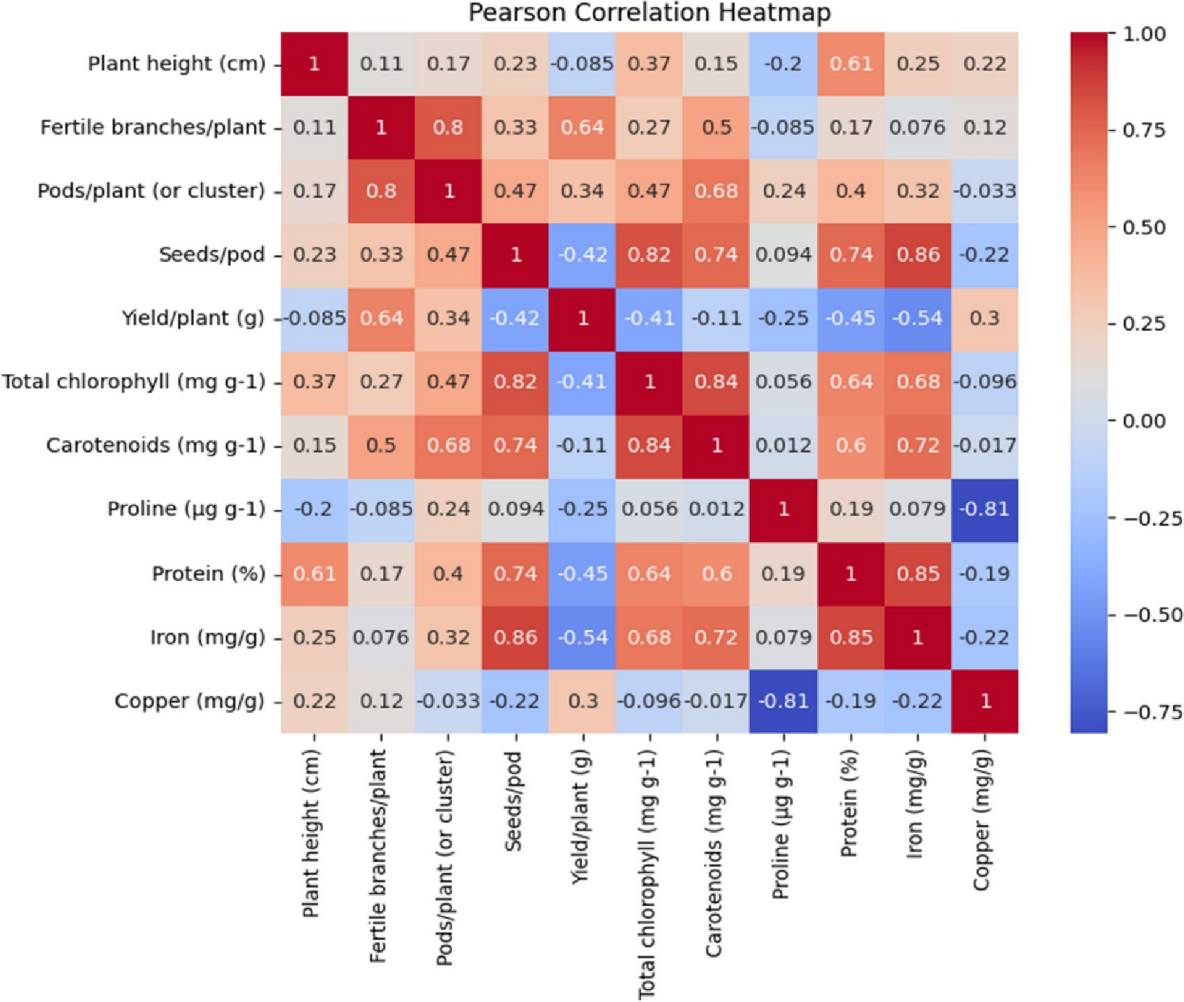
Pearson correlation heatmap between the parameters of both the species. Red color indicates a positive correlation and blue indicates a negative correlation.

The Pearson correlation coefficients were calculated to assess the relationships among the parameters of both the species, and the results were visualized as a heatmap in Fig 8. The Pearson correlation coefficients, which provide insights into the relationships between pairs of the parameters, displayed a range of values spanning from -0.08 to 0.86. Significant positive correlations were observed, with a coefficient of 0.86, between seeds per pod and iron content. In the heatmap, the darker red shades signified strong positive correlations among the parameters, whereas the blue color indicated negative correlations. The presence of strong correlations between iron content and seeds per pod contribute to a plant’s ability to achieve a nutritious high yield potential.

**Fig 9:**
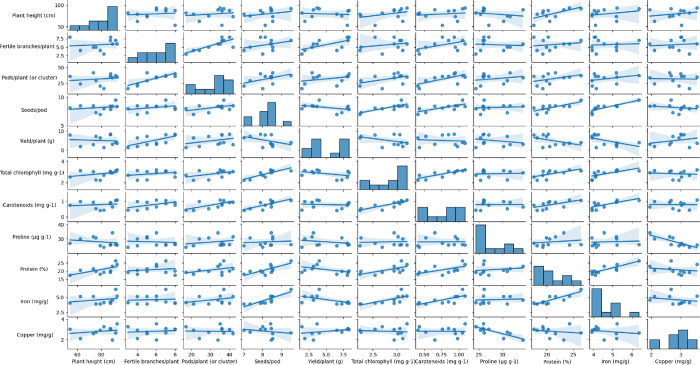
Pairwise scatter plot matrix with linear regression lines between the two variables.

To further explore the linear associations between two continuous variables, a pairwise scatter plot matrix with linear regression lines was created, as depicted in Fig 9. The linear regression lines were fitted to best approximate the data points and represented the relationship between the two variables. This analysis is commonly used to assess the linear association between continuous variables.

In summary, the heatmap and pairwise scatter plot matrix, along with linear regression, offered insights into the correlations and linear associations among the parameters of both PEB and Pusa kasuri. The strong positive correlation among seeds per pod and iron content highlights their potential relationship in the plant’s characteristics.

## Discussion

### High yielding mutant lines

In the M_3_ generation of two fenugreek species, a total of nine mutant lines were identified, each exhibiting significant variability in important yield-attributing traits, such as the number of fertile branches per plant, pods per plant, and overall seed yield. In comparison to the control, these mutant lines demonstrated a notable rise in the average values of these yield-related traits. These results align with the earlier results in lentil [56]. The observed surge in seed yield and other yield- related traits can be attributed to mutagen-induced increased mitotic division, modifications in physiological, biochemical, and metabolic pathways, and interactions with genes regulating yield. The mutants were obtained from lower and intermediate caffeine and SA treatment doses, indicating the potential effectiveness of these mutagenic agents in inducing beneficial changes in yield-related traits. The yield decreases higher concentrations of caffeine and SA in both species might be due chromosomal injuries, failure or restricted pairing, delays in DNA synthesis, or disturbed spindle formation, along with high pollen sterility due to the genotoxic effects of chemical mutagens. The successful isolation of high-yielding mutant lines in the M_3_ generation holds immense promise for crop improvement programs.

In conclusion, the findings underscore the high selection value and significant breeding significance of the identified mutant lines with enhanced yield attributes. The successful application of mutagenic treatments to induce beneficial changes in fenugreek cultivars paves the way for the development of improved varieties that can contribute to increased agricultural productivity and food security.

### Physiological parameters

The present study focused on evaluating chlorophyll and carotenoid contents in the M_3_ high-yielding mutant lines. The results revealed a significant improvement in the mean values of chlorophyll and carotenoid contents in these mutants in contrast to their respective controls. Similar increases in chlorophyll and carotenoid contents have been reported in other studies involving different plant species, such as *Triticum aestivum* [57], *Brassica napus* [58], and *Capsicum annum* [59]. The increase in total chlorophyll and carotenoid contents in the mutants was primarily attributed to the augmentation in chlorophyll-a and β-carotene contents, as documented in previous research [60]. Carotenoids, being natural pigments, play a crucial role in plant health, and their consumption in the human diet is known to offer various health benefits, including their antioxidant properties that help alleviate oxidative stress in the body [61]. The significant improvement in carotenoid contents in the high-yielding mutant lines may be linked to mutagen-induced alterations in the activity levels of carotenoid biosynthesis genes. This up-regulation of carotenoid biosynthesis genes induced by mutagenic treatments might confer tolerance to diverse climate-induced stresses, such as drought and salinity. As a result, it suggests that the identified high-yielding M_3_ mutants may possess tolerance to both biotic and abiotic stresses. These mutants could be further screened in advanced generations after trait stabilization to assess their potential for crop improvement and stress tolerance.

### Seed protein and mineral element contents

Induced mutagenesis is a promising approach for crop improvement, aiming to enhance nutrient contents, particularly protein, in major crops. High-yielding mutant lines were examined for advancements in seed yield and protein content. Some mutants (A, F, J, and L) showed a substantial elevation in overall seed protein content, as reported in various crops by other researchers [56, 62, 63]. The M_3_ high-yielding mutants also exhibited a significant increase in mineral elements like iron (Fe) and copper (Cu), potentially addressing mineral nutrient deficiency. However, there is a risk of merging the improved micronutrient trait with high yield. Tomlekova et al. (2017) [64] also found that higher β-carotene content caused an increase in mineral element content in pepper mutants. It was suggested that increased mineral elements might be due to augmented β-carotene contents in the mutants. These results were confirmed by [65] in faba bean and [66] in chickpea. These findings support the possibility of combining improved yield characteristics with genetic enhancement of micronutrient content to produce mutants with enhanced nutritional profiles. Agronomic biofortification, involving various applications in edible crops, can be an effective method for increasing mineral elements. The development and utilization of mutants with both high yield and high protein content offer promising avenues for advancing agriculture and improving human nutrition. Recently, it has been widely accepted that a series of specific genes regulate protein synthesis in seeds, and changes in proteins are produced due to genetic alterations in most of the events.

### Stomatal behaviour of leaves and micromorphology of seeds

Scanning Electron Microscopy (SEM) has developed as a crucial tool in the field of biology, enabling researchers to explore and analyze the intricate structures of biological specimens with high resolution and detail [67]. Researchers have extensively utilized SEM to study leaf physiology and surface morphology, offering valuable insights into plant adaptations and responses to environmental conditions [68]. SEM analysis revealed a notable increase in stomatal size compared to control plants. This finding has been supported by multiple previous studies [27, 56, 69]. The increase in stomatal size, both in terms of length and width, as well as the number of stomata, has significant implications for the gaseous exchange between the plant tissues and the environment. This enhanced exchange facilitates a more efficient photosynthetic process, leading to an improvement in overall plant yield. The observed changes in stomatal characteristics in high-yielding mutants may be attributed to induced genetic damage and mutations caused by mutagenic agents used in the study.

In addition to stomatal behavior, the research also examined seed morphology and seed coat pattern in various seed mutants of *Trigonella* species. The seed coat pattern or micro-ornamentation on the surface of the outer cell wall can be regarded as having taxonomic value in species identification. However, in the current research, clear differences were detected in the micromorphology of seeds from various mutants of both *Trigonella* species, aligning with findings reported by [70, 71]. The significance of seed sculpturing as a diagnostic character depends on the extent of variation present within and among species, as well as the effectiveness of seed morphology in distinguishing between species at the interspecific level. Cervantes et al. (2016) [72] noted that the integration of digital technologies, along with advancements in quantification and modeling methods, enables a more precise description of seed shape.

In summary, the observed changes in stomatal size and frequency suggest their potential significance in enhancing plant productivity through improved photosynthesis. The investigation of seed micromorphology offers valuable information about the variability in seed traits, which could be of great importance in the context of crop improvement and breeding programs.

### GC-MS analysis

Gas-chromatography coupled with mass spectrometry (GC-MS) is a precise technique for qualitative analysis [73], and in the present study, it was used to identify phytochemical compounds in PEB and Pusa kasuri. Methanol extraction was utilized, a common technique for identifying phytochemicals in various plant species. The study recorded important compounds like linoleic acid, palmitic acid, stearamide, elaidic acid, and dodecanoic acid, among others, which possess pharmaceutical, medicinal, and industrial value. Researchers have conducted GC-MS analysis of fenugreek seeds previously, identifying abundant phytochemicals with health benefits [74–76]. For example, linoleic acid has demonstrated strong anticancer activity, particularly in breast cancer treatment [77]. Hexadecanoic acid, an ester, exhibits various properties such as antioxidant, hypocholesterolemic, pesticide, lubricant, antiandrogenic, flavor, and hemolytic 5-alpha-reductase inhibitor [36]. Other phytochemical constituents like pinene, linoleic acid methyl ester, etc. possess pharmacological activities as antioxidants and are used for antiasthmatic and treatment of sexual and urinary disorders [78]. The compounds identified belong to a diverse range of chemical groups, and a substantial portion of these compounds has been confirmed to possess important biological activities [79]. The significant chemical compounds identified within various extracts are believed to be components of the plants’ defense mechanisms. These compounds can be categorized as protective elements present in the plant, often referred to as ’phytoanticipins’ and ’phytoprotectants’ [80]. Such compounds are of particular interest due to their potential therapeutic applications in treating various diseases and promoting overall health.

### SCoT Marker analysis

SCoT marker analysis is a reliable and versatile DNA marker technique based on the translation initiation site (TIS), which has been successfully validated in rice and peanut [13, 81]. Moreover, the interaction between mutagens and DNA leads to alterations in gene expression and distinctive DNA profiles. Its simplicity and reproducibility have been employed to studies of genetic diversity and taxonomy in diverse plant species like *Nigella sativa* [82], *Mangifera indica* [83], and Common bean [84].

In this study, SCoT marker analysis was utilized to investigate polymorphism in *Trigonella* species. The findings revealed a high abundance of polymorphisms, with 28.3% and 46.7% polymorphic DNA bands observed in PEB and Pusa kasuri, respectively. As per Bhat et al. (2012) [85], genotypic diversity in mutant plants may arise from the emergence of new bands due to mutation and the disappearance of bands due to DNA damage. The informativeness of each primer was assessed using the PIC criteria, and the mean PIC values demonstrated the effectiveness of the marker system. The PIC value ranges from 0 to 1, with values closer to 1 indicating higher levels of polymorphism. Comparable effectiveness of SCoT markers has been reported in other studies involving different crops, with PIC values ranging from 0.10 to 0.76 in *Coffea canephora*, 0.15 to 0.62 in *Laurus nobilis*, 0.31 to 0.39 in *Triticum aestivum*, and 0.43 to 0.47 in chickpea genotypes [86–89]. Earlier research has also highlighted the effectiveness of SCoT markers in evaluating genetic diversity in various species, including *T*. *foenum-graecum* [90], *Papaver bracteatum* [91], and wild salvia [92]. Overall, SCoT marker analysis has proven to be a valuable tool in exploring genetic diversity and polymorphism in different plant species, providing valuable insights for crop improvement and breeding programs.

## Conclusion

The current study highlights the effectiveness of morpho-physiological, biochemical, protein, and mineral content analyses, along with molecular analysis, in selecting fenugreek mutants with varying performance under diverse mutagenic conditions. A broad spectrum of variability was observed across all traits and unique traits that can be harnessed to develop more desirable and resilient crop varieties. Assessment of stomatal and seed characteristics may contribute to enhancing physiological processes and distinguishing between species at the interspecific level, respectively. The presence of pharmaceutical and medicinal compounds in the mutants opens up opportunities for the development of novel herbal remedies for various diseases, potentially resulting in the formulation of new medications. SCoT markers demonstrated efficiency and exhibited stronger discriminating power for the studied species, as evidenced by the high values of genetic diversity indices, polymorphism percentage, and number of specific bands. The findings underscore the ability of mutagens to induce variation in SCoT banding patterns. These bands could be deemed valuable markers in breeding programmes. Cluster analysis segregated the control plants of both species into distinct clusters, confirming that the applied mutagens effectively induced heritable genetic variability in the M_3_ mutants in contrast to their respective controls. Furthermore, it has been established that selection for yield traits can be integrated with genetic enhancement of the nutritional content of fenugreek to potentially generate mutant varieties. Further studies aimed at purification, sequencing and stress-related gene expression analysis of these bands could potentially reduce the expense of breeding programs and serve as a potent strategy for selecting high-yielding mutants.

## Supporting information

S1 File (DOCX)

S1 Raw images (PDF)

## References

[1] Gerland P, Raftery AE, Ševčíková H, Li N, Gu D, Spoorenberg T, et al. World population stabilization unlikely this century. Science. 2014; 346(6206):234–7. doi: 10.1126/science.1257469 25301627 PMC4230924

[2] FAO. The Future of Food and Agriculture: Trends and Challenges; FAO: Rome, Italy, ISBN, 2017; 9789251095515

[3] Chen H, Yada R. Nanotechnologies in agriculture: new tools for sustainable development. Trends in Food Science & Technology. 2011; 22(11):585–94. 10.1016/j.tifs.2011.09.004

[4] Rubiales D, Mikic A. Introduction: legumes in sustainable agriculture. Critical Reviews in Plant Sciences. 2015; 34(1–3):2–3. 10.1080/07352689.2014.897896

[5] Thorat RM, Gaikwad DD. Pharmacognostical and Phytophysicochemical investigations of *Trigonella foenum–graecum* Linn. Journal of Drug Delivery and Therapeutics. 2019; 9(3-s):138–45. 10.22270/jddt.v9i3-s.2810

[6] Aasim M, Baloch FS, Nadeem MA, Bakhsh A, Sameeullah M, Day S. Fenugreek (*Trigonella foenum-graecum* L.): an underutilized edible plant of modern world. Global perspectives on underutilized crops. 2018:381–408. 10.1007/978-3-319-77776-4_12

[7] Bitarafan Z, Asghari HR, Hasanloo T, Gholami A, Moradi F, Khakimov B, et al. The effect of charcoal on medicinal compounds of seeds of fenugreek (*Trigonella foenum-graecum* L.) exposed to drought stress. Industrial Crops and Products. 2019; 131:323–9. 10.1016/j.indcrop.2019.02.003

[8] Wani SA, Kumar P. Fenugreek: A review on its nutraceutical properties and utilization in various food products. Journal of the Saudi Society of Agricultural Sciences. 2018; 17(2):97–106. 10.1016/j.jssas.2016.01.007

[9] Al-Jasass FM, Al-Jasser MS. Chemical composition and fatty acid content of some spices and herbs under Saudi Arabia conditions. The scientific world journal. 2012; 2012:1–5. doi: 10.1100/2012/859892 23319888 PMC3540753

[10] Yadav R, Khare RK, Singhal A. Qualitative phytochemical screening of some selected medicinal plants of shivpuri district (MP). Int. J. Life. Sci. Scienti. Res. 2017; 3(1):844–7.

[11] Fan S, Chang J, Zong Y, Hu G, Jia J. GC-MS analysis of the composition of the essential oil from Dendranthema indicum Var. Aromaticum using three extraction methods and two columns. Molecules. 2018;23(3):576. doi: 10.3390/molecules23030576 29510531 PMC6017652

[12] Sufyan M, Badshah I, Ahmad M, Zafar M, Bahadur S, Rashid N. Identification of medicinally used Flora using pollen features imaged in the scanning electron microscopy in the lower Margalla Hills Islamabad Pakistan. Microscopy and Microanalysis. 2018; 24(3):292–9. doi: 10.1017/S1431927618000326 29952283

[13] Collard BC, Mackill DJ. Start codon targeted (SCoT) polymorphism: a simple, novel DNA marker technique for generating gene-targeted markers in plants. Plant molecular biology reporter. 2009; 27:86–93. 10.1007/s11105-008-0060-5

[14] Etminan A, Pour-Aboughadareh A, Mohammadi R, Ahmadi-Rad A, Noori A, Mahdavian Z, et al. Applicability of start codon targeted (SCoT) and inter-simple sequence repeat (ISSR) markers for genetic diversity analysis in durum wheat genotypes. Biotechnology & Biotechnological Equipment. 2016; 30(6):1075–81. 10.1080/13102818.2016.1228478

[15] Mirzahosein-Tabrizi M, Ghanavati F, Azizinezhad R, Etminan A. Genetic diversity revealed by phytochemical and molecular analyses among and within eight *Trigonella* sp. Journal of Crop Science and Biotechnology. 2023; 26(3):345–57. 10.1007/s12892-022-00183-z

[16] Hamidi H, Talebi R, Keshavarzi F. Comparative efficiency of functional gene-based markers, start codon targeted polymorphism (SCoT) and conserved DNA-derived polymorphism (CDDP) with ISSR markers for diagnostic fingerprinting in wheat (*Triticum aestivum* L.). Cereal research communications. 2014; 42(4):558–67. 10.1556/crc.2014.0010

[17] Que Y, Pan Y, Lu Y, Yang C, Yang Y, Huang N, et al. Genetic analysis of diversity within a Chinese local sugarcane germplasm based on start codon targeted polymorphism. BioMed research international. 2014; 2014:1–10. doi: 10.1155/2014/468375 24779012 PMC3980922

[18] Ibrahim SD, Adawy SS, Atia MA, Alsamman AM, Mokhtar MM. Genetic diversity, variety identification and gene detection in some Egyptian grape varieties by SSR and SCoT markers. Plant Omics. 2016; 9(5).

[19] Abd El-Moneim D, ELsarag EI, Aloufi S, El-Azraq AM, ALshamrani SM, Safhi FA, et al. Quinoa (Chenopodium quinoa Willd.): genetic diversity according to ISSR and SCoT markers, relative gene expression, and morpho-physiological variation under salinity stress. Plants. 2021; 10(12):2802. doi: 10.3390/plants10122802 34961273 PMC8707205

[20] El-Mahdy MT, Youssef M, Elazab DS. In vitro screening for salinity tolerance in pomegranate (Punica granatum L.) by morphological and molecular characterization. Acta Physiologiae Plantarum. 2022; 44(2):27. 10.1007/s11738-022-03361-2

[21] Agarwal A, Gupta V, Haq SU, Jatav PK, Kothari SL, Kachhwaha S. Assessment of genetic diversity in 29 rose germplasms using SCoT marker. Journal of King Saud University-Science. 2019; 31(4):780–8. 10.1016/j.jksus.2018.04.022

[22] Mackinney G. Absorption of light by chlorophyll solutions. Journal of biological chemistry. 1941; 140(2):315–22. 10.1016/S0021-9258(18)51320-X

[23] Arnon DI. Copper enzymes in isolated chloroplasts. Polyphenoloxidase in Beta vulgaris. Plant physiology. 1949; 24(1):1. doi: 10.1104/pp.24.1.1 16654194 PMC437905

[24] Bates LS, Waldren RP, Teare ID. Rapid determination of free proline for water-stress studies. Plant and soil. 1973; 39:205–7. 10.1007/BF00018060

[25] Lowry OH, Rosebrough NJ, Farr AL, Randall RJ. Protein measurement with the Folin phenol reagent. J biol Chem. 1951; 193(1):265–75. 14907713

[26] Gupta PK. Plant analysis, In: Plant Soil, Water and Fertilizer Analysis. Agrobios. 2004; pp 252–292.

[27] Naaz N, Choudhary S, Sharma N, Hasan N, Al Shaye NA, Abd El-Moneim D. Frequency and spectrum of M2 mutants and genetic variability in cyto-agronomic characteristics of fenugreek induced by caffeine and sodium azide. Frontiers in Plant Science. 2023; 13:1030772. doi: 10.3389/fpls.2022.1030772 36726682 PMC9886007

[28] Doyle JJ, Doyle JL. A rapid DNA isolation procedure for small quantities of fresh leaf tissue. Phytochemical bulletin. 1987; 19:11–15

[29] Sambrook J, Fritsch EF, Maniatis T. Molecular cloning: a laboratory manual. Cold spring harbor laboratory press. 1989.

[30] Sneath PH. The principles and practice of numerical classification. Numerical taxonomy. 1973;573.

[31] Sasaki K, Hayashi K, Matsuya Y, Sugimoto K, Lee JB, Kurosaki F, et al. In vitro and in vivo antiherpetic effects of (1 R, 2 R)-1-(5′-methylful-3′-yl) propane-1, 2, 3-triol. Journal of natural medicines. 2016; 70:217–24. doi: 10.1007/s11418-016-0964-6 26763002

[32] Sakika KA, Saiman MZ, Zamakshshari NH, Ahmed IA, Nasharuddin MN, Hashim NM. Analysis of Antioxidant Properties and Volatile Compounds of Honeys from Different Botanical and Geographical Origins. Sains Malaysiana. 2022; 51(4):1111–21. 10.17576/jsm-2022-5104-13

[33] Zhou B, Yuan X, Fan L, Pan Z, Chang X, Jiang S, et al. Synthesis and antifungal activities of novel trifluoroethane derivatives with coumarin, indole and thiophene. Journal of Saudi Chemical Society. 2022; 26(6):101572. 10.1016/j.jscs.2022.101572

[34] Wei LS, Wee W, Siong JY, Syamsumir DF. Characterization of anticancer, antimicrobial, antioxidant properties and chemical compositions of Peperomia pellucida leaf extract. Acta Medica Iranica. 2011:670–4. 22071643

[35] Shaaban MT, Ghaly MF, Fahmi SM. Antibacterial activities of hexadecanoic acid methyl ester and green‐synthesized silver nanoparticles against multidrug‐resistant bacteria. Journal of basic microbiology. 2021; 61(6):557–68. doi: 10.1002/jobm.202100061 33871873

[36] Shaheed KA, AlGaraawi NI, Alsultany AK, Abbas ZH, Khshayyish IK, Al Khazali MT. Analysis of bioactive phytochemical compound of (Cyperus iria L.) By using gas chromatography–mass spectrometry. InIOP Conference Series: Earth and Environmental Science 2019 Nov 1 (Vol. 388, No. 1, p. 012064). IOP Publishing. doi: 10.1088/1755-1315/388/1/012064

[37] Aparna V, Dileep KV, Mandal PK, Karthe P, Sadasivan C, Haridas M. Anti‐inflammatory property of n‐hexadecanoic acid: structural evidence and kinetic assessment. Chemical biology & drug design. 2012; 80(3):434–9. doi: 10.1111/j.1747-0285.2012.01418.x 22642495

[38] Dhar Dubey KK, Sharma G, Kumar A. Conjugated linolenic acids: implication in cancer. Journal of agricultural and food chemistry. 2019; 67(22):6091–101. doi: 10.1021/acs.jafc.9b01379 31070027

[39] Ghavam M, Afzali A, Manca ML. Chemotype of damask rose with oleic acid (9 octadecenoic acid) and its antimicrobial effectiveness. Scientific reports. 2021; 11(1):8027. doi: 10.1038/s41598-021-87604-1 33850230 PMC8044169

[40] Adnan M, Nazim Uddin Chy M, Mostafa Kamal AT, Azad MO, Paul A, Uddin SB, et al. Investigation of the biological activities and characterization of bioactive constituents of *Ophiorrhiza rugosa* var. prostrata (D. Don) & Mondal leaves through in vivo, in vitro, and in silico approaches. Molecules. 2019; 24(7):1367. 10.3390/molecules24071367 30965575 PMC6480688

[41] Jalalvand AR, Zhaleh M, Goorani S, Zangeneh MM, Seydi N, Zangeneh A, et al. Chemical characterization and antioxidant, cytotoxic, antibacterial, and antifungal properties of ethanolic extract of *Allium Saralicum* RM Fritsch leaves rich in linolenic acid, methyl ester. Journal of Photochemistry and Photobiology B: Biology. 2019; 192:103–12. 10.1016/j.jphotobiol.2019.01.017 30731424

[42] Jain NK, Tailang M, Kumar S, Chandrasekaran B, Alghazwani Y, Chandramoorthy HC, et al. Appraising the therapeutical potentials of *Alchornea laxiflora* (Benth.) Pax & K. Hoffm., an underexplored medicinal herb: A systematic review. Frontiers in Pharmacology. 2022; 13:958453. 10.3389/fphar.2022.958453 36545314 PMC9761395

[43] Kannaiyan SK, Bagthasingh C, Vetri V, Aran SS, Venkatachalam K. Nutritional, textural and quality attributes of white and dark muscles of little tuna (*Euthynnus affinis*).

[44] Ghosh K, Adak A, Halder SK, Mondal KC. Physicochemical characteristics and lactic acid bacterial diversity of an ethnic rice fermented mild alcoholic beverage, haria. Frontiers in Sustainable Food Systems. 2021; 5:680738. 10.3389/fsufs.2021.680738

[45] Yakubu Y, Lee SY, Shaari K. Chemical Profiles of Terminalia catappa LINN Nut and Terminalia subspathulata KING Fruit. Pertanika Journal of Tropical Agricultural Science. 2021; 44(4). doi: 10.47836/pjtas.44.4.06

[46] Fayyaz M, Akbar M, Iqbal MS, Ahsan T, Wu YH, Khalil T. Characterization of antifungal molecules of Calotropis procera against Fusarium oxysporum, the causal agent of Fusarium wilt in crops.

[47] Gahtori R, Tripathi AH, Chand G, Pande A, Joshi P, Rai RC, et al. Phytochemical Screening of Nyctanthes arbor-tristis Plant Extracts and Their Antioxidant and Antibacterial Activity Analysis. Applied Biochemistry and Biotechnology. 2024; 196(1):436–56. doi: 10.1007/s12010-023-04552-4 37140779

[48] Kosiakova H, Berdyshev A, Dosenko V, Drevytska T, Herasymenko O, Hula N. The Involvement of PPARγ In Anti-Inflammatory Activity of N-Stearoylethanolamide. 10.21203/rs.3.rs-636781/v1 PMC9641209 36387464

[49] Loffredo L, Perri L, Di Castelnuovo A, Iacoviello L, De Gaetano G, Violi F. Supplementation with vitamin E alone is associated with reduced myocardial infarction: A meta-analysis. Nutrition, Metabolism and Cardiovascular Diseases. 2015; 25(4):354–63. doi: 10.1016/j.numecd.2015.01.008 25779938

[50] Meshram A, Srivastava N, Bhagyawant SS. Identification of phytoconstituents present in Epipremnum aureum (linden and andre) g S bunting by GC-MS. Int J Life Sci Rev. 2016;2(3):45–51.

[51] Lee GY, Han SN. The role of vitamin E in immunity. Nutrients. 2018; 10(11):1614. doi: 10.3390/nu10111614 30388871 PMC6266234

[52] Hager TE. GC-MS analysis, Antimicrobial and antioxidant Activity of Fixed Oil from Vangueria magdascariensis (L) Seeds:(Sudanese Pew). مجلة كردفان للعلوم التربوية والإنسانية. 2020;1(1):135–43.‎

[53] Atta-Ur-Rahman, Sultana N, Shahwar D, Choudhary MI. Two new fatty esters from Rhazya stricta roots (Apocynanaceae). Natural Product Research. 2008; 22(15):1350–4. doi: 10.1080/14786410701712897 19023793

[54] Sianipar NF, Purnamaningsih R. Bioactive compounds of fourth generation gamma-irradiated Typhoniumflagelliforme Lodd. mutants based on gas chromatography-mass spectrometry. InIOP Conference Series: Earth and Environmental Science 2016 Aug 1 (Vol. 41, No. 1, p. 012025). IOP Publishing. doi: 10.1088/1755-1315/41/1/012025

[55] Palmer BD, Rewcastle GW, Atwell GJ, Baguley BC, Denny WA. Potential antitumor agents. 54. Chromophore requirements for in vivo antitumor activity among the general class of linear tricyclic carboxamides. Journal of medicinal chemistry. 1988; 31(4):707–12. doi: 10.1021/jm00399a003 3351846

[56] Shahwar D, Ansari MY, Park Y. Physio-biochemical analysis and molecular characterization of induced lentil mutant lines. Plos one. 2022;17(10):e0274937. doi: 10.1371/journal.pone.0274937 36279277 PMC9591049

[57] Borzouei A, Kafi M, Khazaei H, Naseriyan B, Majdabadi A. Effects of gamma radiation on germination and physiological aspects of wheat (*Triticum aestivum* L.) seedlings. Pak. J. Bot. 2010; 42(4):2281–90.

[58] Chen W, Wan S, Shen L, Zhou Y, Huang C, Chu P, et al. Histological, physiological, and comparative proteomic analyses provide insights into leaf rolling in *Brassica napus*. Journal of proteome research. 2018; 17(5):1761–72. 10.1021/acs.jproteome.7b00744 29693398

[59] Hasan N, Choudhry S, Laskar RA. Studies on qualitative and quantitative characters of mutagenised chili populations induced through MMS and EMS. Vegetos. 2020; 33(4):793–9. 10.1007/s42535-020-00164-z

[60] Tomlekova N, Todorova V, Petkova V, Yancheva S, Nikolova V, Panchev I, et al. Creation and evaluation of induced mutants and valuable tools for pepper breeding programmes. Induced Plant Mutations in the Genomics Era, Rome, Italy: Food and Agriculture Organization of the United Nations. 2009; 1:187–90.

[61] Fiedor J, Burda K. Potential role of carotenoids as antioxidants in human health and disease. Nutrients. 2014; 6(2):466–88. doi: 10.3390/nu6020466 24473231 PMC3942711

[62] Laskar RA, Laskar AA, Raina A, Khan S, Younus H. Induced mutation analysis with biochemical and molecular characterization of high yielding lentil mutant lines. International journal of biological macromolecules. 2018; 109:167–79. doi: 10.1016/j.ijbiomac.2017.12.067 29248554

[63] Raina A, Laskar RA, Tantray YR, Khursheed S, Wani MR, Khan S. Characterization of induced high yielding cowpea mutant lines using physiological, biochemical and molecular markers. Scientific reports. 2020; 10(1):3687. doi: 10.1038/s41598-020-60601-6 32111942 PMC7048850

[64] Tomlekova NB, White PJ, Thompson JA, Penchev EA, Nielen S. Mutation increasing β-carotene concentrations does not adversely affect concentrations of essential mineral elements in pepper fruit. PloS one. 2017; 12(2):e0172180. 10.1371/journal.pone.0172180 28207797 PMC5313226

[65] Steen JE, Peoples Mark B, Henrik HN. Faba bean in cropping systems. Field Crops Research. 2010;115(3). doi: 10.1016/j.fcr.2009.10.008

[66] Kozgar MI, Samiullah Khan SK, Wani MR. Variability and correlations studies for total iron and Manganese contents of chickpea (*Cicer arietinum* L.) high yielding mutants. 2012; 7(7):437–44

[67] Mohammed A, Abdullah A. Scanning electron microscopy (SEM): A review. InProceedings of the 2018 International Conference on Hydraulics and Pneumatics—HERVEX, Băile Govora, Romania 2018; (Vol. 2018, pp. 7–9).

[68] Caldwell D, Iyer-Pascuzzi AS. A scanning electron microscopy technique for viewing plant− microbe interactions at tissue and cell-type resolution. Phytopathology. 2019; 109(7):1302–11. doi: 10.1094/PHYTO-07-18-0216-R 30694115

[69] Mallick M, Awasthi OP, Paul V, Verma MK, Jha G. Effect of physical and chemical mutagens on leaf sclerophylly and stomatal characteristics of *Kinnow mandarin* mutants. Indian Journal of Horticulture. 2016;73(2):291–3. doi: 10.5958/0974-0112.2016.00063.3

[70] Çeter T, Pinar NM, Akan H, Ekici M, Aytaç Z. Comparative seed morphology of *Trigonella* L. species (Leguminosae) in Turkey. Afr J Agric Res. 2012; 7(3):509–22. doi: 10.5897/AJAR11.1528

[71] Turki Z, El-Shayeb F, Abozeid A. Seed morphology of some *Trigonella* L. species (Fabaceae) and its taxonomic significance. International Journal of Science and Research. 2013; 3(12):940–8.

[72] Cervantes E, Martín JJ, Saadaoui E. Updated methods for seed shape analysis. Scientifica. 2016; 2016. doi: 10.1155/2016/5691825 27190684 PMC4846768

[73] Cong Z, Meiling Q, Qinglong S. GC-MS analysis and antibacterial activity of Piper Beetle Linn leaves against Streptococcus mutans. J Pharm Biomed Anal. 2007;44:464–70.17306492

[74] Qadir A, Khan N, Arif M, Warsi MH, Ullah SN, Yusuf M. GC–MS analysis of phytoconstituents present in Trigonella foenumgraecum L. seeds extract and its antioxidant activity. Journal of the Indian Chemical Society. 2022; 99(6):100503. 10.1016/j.jics.2022.100503

[75] Kiashi F, Momeni Nasab F, Tavakoli S, Aghaahmadi M, Goodarzi S, Pirali Hamedani M, et al. Trigonella teheranica: a valuable source of phytochemicals with antibacterial, antioxidant and cytotoxic properties. Natural Product Research. 2022 Dec 17;36(24):6405–9. doi: 10.1080/14786419.2022.2032694 35073800

[76] Alabi MA, Arowolo MA, Na’Allah A, Prabhkar PK, Linus EG, Aransiola SA, et al. Phytochemicals and anticancer activity of methanol extract of Trigonella foenum-greacum seed on breast cancer cell lines. South African Journal of Botany. 2023 Sep 1;160:273–81. 10.1016/j.sajb.2023.07.021

[77] Iqbal J, Abbasi BA, Mahmood T, Kanwal S, Ali B, Shah SA, et al. Plant-derived anticancer agents: A green anticancer approach. Asian Pacific Journal of Tropical Biomedicine. 2017;7(12):1129–50. 10.1016/j.apjtb.2017.10.016

[78] Costanzo A, Panseri S, Giorgi A, Romano A, Caprioli M, Saino N. The odour of sex: sex-related differences in volatile compound composition among barn swallow eggs carrying embryos of either sex. PLoS One. 2016;11(11):e0165055. doi: 10.1371/journal.pone.0165055 27851741 PMC5112806

[79] Ruthiran P, Selvaraj CI. Phytochemical screening and in vitro antioxidant activity of Parkia timoriana (DC.) Merr. Research Journal of Biotechnology Vol. 2017;12:12.

[80] Salehi B, Ata A, V. Anil Kumar N, Sharopov F, Ramírez-Alarcón K, Ruiz-Ortega A, et al. Antidiabetic potential of medicinal plants and their active components. Biomolecules. 2019;9(10):551. doi: 10.3390/biom9100551 31575072 PMC6843349

[81] Xiong FQ, Tang RH, Chen ZL, Pan LH, Zhuang WJ. SCoT: a novel gene targeted marker technique based on the translation start codon. Mol Plant Breed. 2009;7(3):635–8.

[82] Golkar P, Nourbakhsh V. Analysis of genetic diversity and population structure in Nigella sativa L. using agronomic traits and molecular markers (SRAP and SCoT). Industrial Crops and Products. 2019;130:170–8. 10.1016/j.indcrop.2018.12.074

[83] Zhou L, He XH, Yu HX, Chen MY, Fan Y, Zhang XJ, et al. Evaluation of the genetic diversity of mango (Mangifera indica L.) seedling germplasm resources and their potential parents with start codon targeted (SCoT) markers. Genetic Resources and Crop Evolution. 2020;67:41–58. 10.1007/s10722-019-00865-8

[84] Yeken MZ, Emiralioğlu O, Çiftçi V, Bayraktar H, Palacioğlu G, Özer G. Analysis of genetic diversity among common bean germplasm by start codon targeted (SCoT) markers. Molecular Biology Reports. 2022;49(5):3839–47. doi: 10.1007/s11033-022-07229-z 35301653

[85] Bhat TM, Ansari MY, Alka, Aslam R. Sodium azide (NaN3) induced genetic variation of Psoralea corylifolia L. and analysis of variants using RAPD markers. The Nucleus. 2012;55(3):149–54. 10.1007/s13237-012-0069-x

[86] Hajibarat Z, Saidi A, Hajibarat Z, Talebi R. Characterization of genetic diversity in chickpea using SSR markers, start codon targeted polymorphism (SCoT) and conserved DNA-derived polymorphism (CDDP). Physiology and molecular biology of plants. 2015;21:365–73. doi: 10.1007/s12298-015-0306-2 26261401 PMC4524857

[87] Huded AK, Jingade P, Bychappa M, Mishra MK. Genetic diversity and population structure analysis of coffee (Coffea canephora) germplasm collections in Indian Gene Bank employing SRAP and SCoT markers. International journal of fruit science. 2020;20(sup2):S757–84. 10.1080/15538362.2020.1768618

[88] Ghobadi G, Etminan A, Mehrabi AM, Shooshtari L. Molecular diversity analysis in hexaploid wheat (Triticum aestivum L.) and two Aegilops species (Aegilops crassa and Aegilops cylindrica) using CBDP and SCoT markers. Journal of Genetic Engineering and Biotechnology. 2021;19(1):56. doi: 10.1186/s43141-021-00157-8 33852105 PMC8046865

[89] Yilmaz A, Ciftci V. Genetic relationships and diversity analysis in Turkish laurel (Laurus nobilis L.) germplasm using ISSR and SCoT markers. Molecular Biology Reports. 2021;48(5):4537–47. doi: 10.1007/s11033-021-06474-y 34148209

[90] Daneshmand H, Etminan AR, Qaderi A. Diversity evaluation of *Trigonella foenum-graecum* populations using DNA markers and phytochemical characteristics. Journal of Medicinal Plants. 2017;16(63):119–32.

[91] Qaderi A, Omidi M, Pour-Aboughadareh A, Poczai P, Shaghaghi J, Mehrafarin A, et al. Molecular diversity and phytochemical variability in the Iranian poppy (Papaver bracteatum Lindl.): A baseline for conservation and utilization in future breeding programmes. Industrial crops and products. 2019;130:237–47. 10.1016/j.indcrop.2018.12.079

[92] Etminan A, Pour-Aboughadareh A, Noori A, Ahmadi-Rad A, Shooshtari L, Mahdavian Z, et al. Genetic relationships and diversity among wild Salvia accessions revealed by ISSR and SCoT markers. Biotechnology & Biotechnological Equipment. 2018; 32(3):610–7. 10.1080/13102818.2018.1447397

