# Supplementary material for: Biochemical and molecular profiling of induced high yielding M3 mutant lines of two Trigonella species: Insights into improved yield potential
Source: PLoS One. 2024 Jul 29;19(7):e0305691. doi: 10.1371/journal.pone.0305691 (PMC11285971; doi:10.1371/journal.pone.0305691)
Supplement: S1 File — (DOCX) [file pone.0305691.s001.docx]

**S1 Table:** Morphological description of *T. foenum-graecum* and *T. corniculata*.

| S.No. | Morphological features | *Trigonella foenum-graecum*  (PEB) | *Trigonella corniculata*  (Pusa kasuri) |
| --- | --- | --- | --- |
| 1 | Plant height | Erect (85-95cm) | Erect to creeping (75-80cm) |
| 2 | Growth habit | Normal branching | Bushy |
| 3 | Leaves | Trifoliate, oblanceolate to oblong | Trifoliate, obovate to oblong-wedge-shaped |
| 4 | Flower colour | White to yellowish | Yellow |
| 5 | Pod | Sword-shaped | Sickle-shaped |
| 6 | Seed shape | Cuboid | Elliptical |
| 7 | Seed colour | Golden-yellowish | Yellowish |

**S2 Table:** Various phytochemical compounds detected in the high yielding M_3_ mutants of *T. foenum-graecum*.

|  | Phytochemicals | C_1_  (PEB) | | Mutant A | | Mutant B | | Mutant C | | Mutant F | | Mutant G | | |
| --- | --- | --- | --- | --- | --- | --- | --- | --- | --- | --- | --- | --- | --- | --- |
|  |  | R.Time | Area % | R.Time | Area % | R.Time | Area % | R.Time | Area % | R.Time | Area % | R.Time | Area % |  |
| 1 | Glycerin | - | - | - | - | 5.287 | 1.90 | 5.264 | 5.21 | - | - | 5.316 | 3.53 |  |
| 2 | Cyclohexanamine, N-methyl | 11.803 | 7.36 | 11.796 | 5.64 | - | - | - | - | 11.793 | 5.08 | - | - |  |
| 3 | 1H-Azepin-1-amine, hexahydro- | 12.840 | 12.47 | 12.861 | 18.85 | 13.364 | 1.69 | - | - | 12.851 | 17.47 | 13.346 | 1.26 |  |
| 4 | .delta.-Dodecalactone | 14.726 | 1.33 | 14.716 | 2.02 | 21.447 | 3.28 | - | - | 14.727 | 1.98 | - | - |  |
| 5 | 2-Naphthalenol, decahydro- | 24.026 | 2.09 | 24.031 | 3.61 | 32.669 | 12.88 | 32.663 | 13.74 | 24.033 | 3.15 | 32.686 | 13.49 |  |
| 6 | 3-Nitro-4-hydroxypyridine | 25.356 | 0.18 | - | - | 34.359 | 5.34 | 34.373 | 4.53 | - | - | - | - |  |
| 7 | 1H-Azepine, hexahydro-3,3,5-trimethyl- | - | - | - | - | 42.207 | 5.11 | 42.205 | 3.85 | - | - | 42.232 | 5.12 |  |
| 8 | Hexadecanoic acid, methyl ester | 33.034 | 1.11 | 33.037 | 1.47 | 43.851 | 2.42 | 43.845 | 2.53 | 33.045 | 2.15 | 43.855 | 2.05 |  |
| 9 | n-Hexadecanoic acid | 33.993 | 2.57 | 33.956 | 1.33 | 44.885 | 6.42 | 44.869 | 7.13 | 33.955 | 1.12 | 44.898 | 5.02 |  |
| 10 | 9,12-Octadecadienoic acid (Z,Z)-, meth | 36.418 | 2.96 | 36.421 | 3.93 | 48.020 | 5.20 | 48.008 | 5.37 | 36.432 | 5.94 | 48.025 | 4.18 |  |
| 11 | 9-Octadecenoic acid, methyl ester, (E)- | 36.543 | 2.33 | 36.548 | 3.48 | 48.186 | 3.57 | 48.181 | 4.58 | 36.560 | 5.74 | 48.195 | 3.87 |  |
| 12 | Octadecanamide, N-(2-hydroxyethyl)- | 37.345 | 5.43 | 37.347 | 3.84 | 55.054 | 9.09 | 55.063 | 9.10 | 37.349 | 2.27 | 49.270 | 14.12 |  |
| 13 | 9,12-Octadecadienoic acid (Z,Z)- | 37.437 | 3.43 | - | - | 49.152 | 6.54 | 49.125 | 7.63 | - | - | 49.179 | 5.51 |  |
| 14 | Cis-9-hexadecenal | - | - | 37.472 | 3.15 | - | - | 49.285 | 12.95 | - | - | - | - |  |
| 15 | 9-Tetradecenal, (Z)- | 37.530 | 5.72 | - | - | - | - | - | - | 37.469 | 3.25 | 49.341 | 6.80 |  |
| 16 | 2-Pyrrolidinone, 1-(9-octadecenyl)- | 40.616 | 1.72 | 40.607 | 1.15 | - | - | - | - | 40.612 | 0.82 | - | - |  |
| 17 | Vitamin E | - | - | - | - | 53.964 | 7.02 | - | - | - | - | 53.974 | 5.92 |  |
| 18 | Hexadecanoic acid, 2-hydroxy-1-(hydro | 43.955 | 5.46 | 43.956 | 5.91 | 58.691 | 6.44 | 58.677 | 8.55 | 43.963 | 5.68 | 58.705 | 9.73 |  |
| 19 | 6,9-Octadecadienoic acid, methyl ester | 46.773 | 3.58 | 46.776 | 4.41 | - | - | - | - | 46.787 | 4.03 | - | - |  |
| 20 | 9-Octadecenoic acid (Z)-, 2,3-dihydroxy | 46.841 | 8.12 | 46.839 | 8.73 | - | - | - | - | 46.844 | 8.42 | - | - |  |
| 21 | Octadecanoic acid, 2,3-dihydroxypropyl e | 47.213 | 3.71 | 47.212 | 4.28 | - | - | - | - | 47.220 | 3.59 | - | - |  |
| 22 | Stigmast-5-en-3-ol, oleate | 55.994 | 5.62 | 56.001 | 7.35 | - | - | - | - | 56.014 | 6.73 | - | - |  |

**S3 Table:** Various phytochemical compounds detected in the high yielding M_3_ mutants of *T. corniculata*.

| Phytochemicals | C_2_  (Pusa kasuri) | | Mutant J | | Mutant L | | Mutant N | | Mutant O | |
| --- | --- | --- | --- | --- | --- | --- | --- | --- | --- | --- |
|  | R.Time | Area % | R.Time | Area % | R.Time | Area % | R.Time | Area % | R.Time | Area % |
| Glycerin | - | - | - | - | 5.267 | 6.32 | 5.267 | 3.41 | 5.246 | 6.49 |
| 2,4-Dihydroxy-2,5-dimethyl-3(2H)-furan | - | - | - | - | 12.404 | 2.51 | 12.391 | 2.16 | 12.411 | 2.83 |
| 2-Methoxythiophene | 12.642 | 0.61 | 12.687 | 1.74 | - | - | - | - | - | - |
| Cyclohexanamine, N-methyl- | - | - | 12.848 | 3.09 | - | - | - | - | - | - |
| .delta.-Dodecalactone | 14.710 | 0.99 | 14.720 | 1.99 | - | - | - | - | - | - |
| 2-Naphthalenol, decahydro- | 24.021 | 0.16 | 24.027 | 0.76 | 32.664 | 2.48 | 32.656 | 3.36 | 32.655 | 6.07 |
| Hexadecanoic acid, methyl ester | 33.043 | 1.19 | 33.047 | 2.16 | 43.844 | 2.45 | 43.848 | 2.10 | 43.845 | 2.85 |
| n-Hexadecanoic acid | 34.003 | 5.11 | 33.959 | 2.49 | 44.876 | 11.63 | 44.886 | 11.69 | 44.862 | 7.40 |
| 9,12-Octadecadienoic acid (Z,Z)-, methy | 36.426 | 3.32 | 36.428 | 5.28 | 48.019 | 5.12 | 48.016 | 4.39 | 48.010 | 5.98 |
| 9-Octadecenoic acid, methyl ester, (E)- | - | - | - | - | 48.183 | 4.14 | 48.182 | 3.64 | 48.180 | 3.92 |
| 9,12,15-Octadecatrienoic acid, methyl e | - | - | 36.548 | 3.13 | - | - | - | - | - | - |
| 9,12-Octadecadienoyl chloride, (Z,Z)- | 36.553 | 1.66 | - | - | - | - | - | - | 49.276 | 12.41 |
| 9,12-Octadecadienoic acid (Z,Z)- | 37.415 | 5.23 | - | - | 49.136 | 12.47 | 49.150 | 11.21 | 49.121 | 9.25 |
| 13-Hexyloxacyclotridec-10-en-2-one | - | - | 37.350 | 4.81 | - | - | - | - | - | - |
| 9-Tetradecenal, (Z)- | - | - | - | - | 49.297 | 16.40 | - | - | - | - |
| cis-9-Hexadecenal | 37.515 | 5.00 | 37.461 | 4.44 | - | - | - | - | - | - |
| Octadecanamide, N-(2-hydroxyethyl)- | 40.621 | 11.95 | 40.498 | 2.40 | 54.476 | 7.29 | 54.475 | 7.38 | 54.464 | 8.77 |
| Carbamic acid, 2-(dimethylamino)ethyl | 42.997 | 2.38 | 42.896 | 3.93 | - | - | - | - | - | - |
| Bis(2-(Dimethylamino)ethyl) ether | - | - | 43.427 | 0.16 | 57.396 | 3.52 | 57.404 | 3.50 | 57.390 | 3.35 |
| Hexadecanoic acid, 2-hydroxy-1-(hydro | 44.107 | 11.55 | 44.093 | 14.49 | 58.683 | 14.26 | 58.685 | 7.15 | 58.675 | 7.56 |


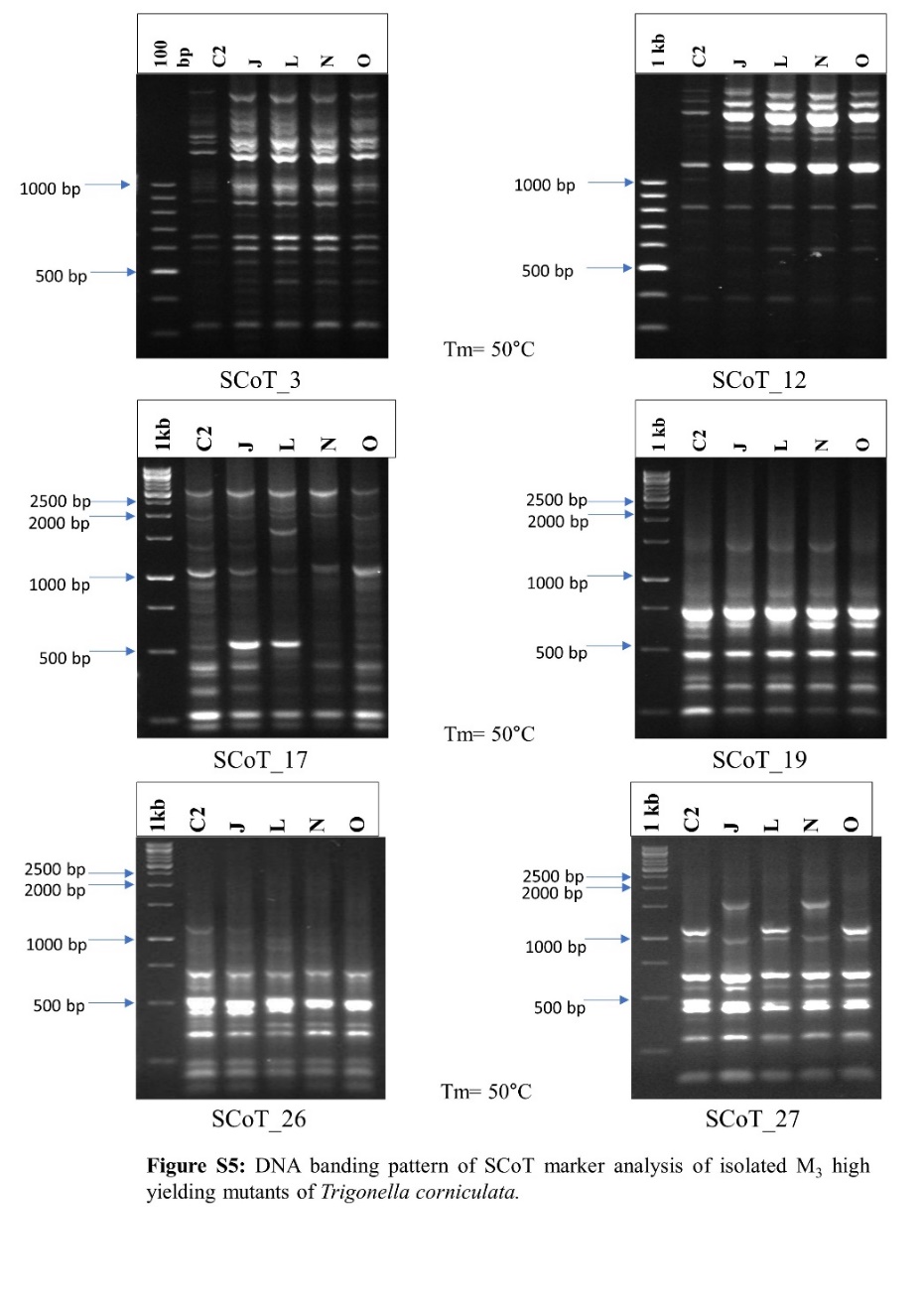

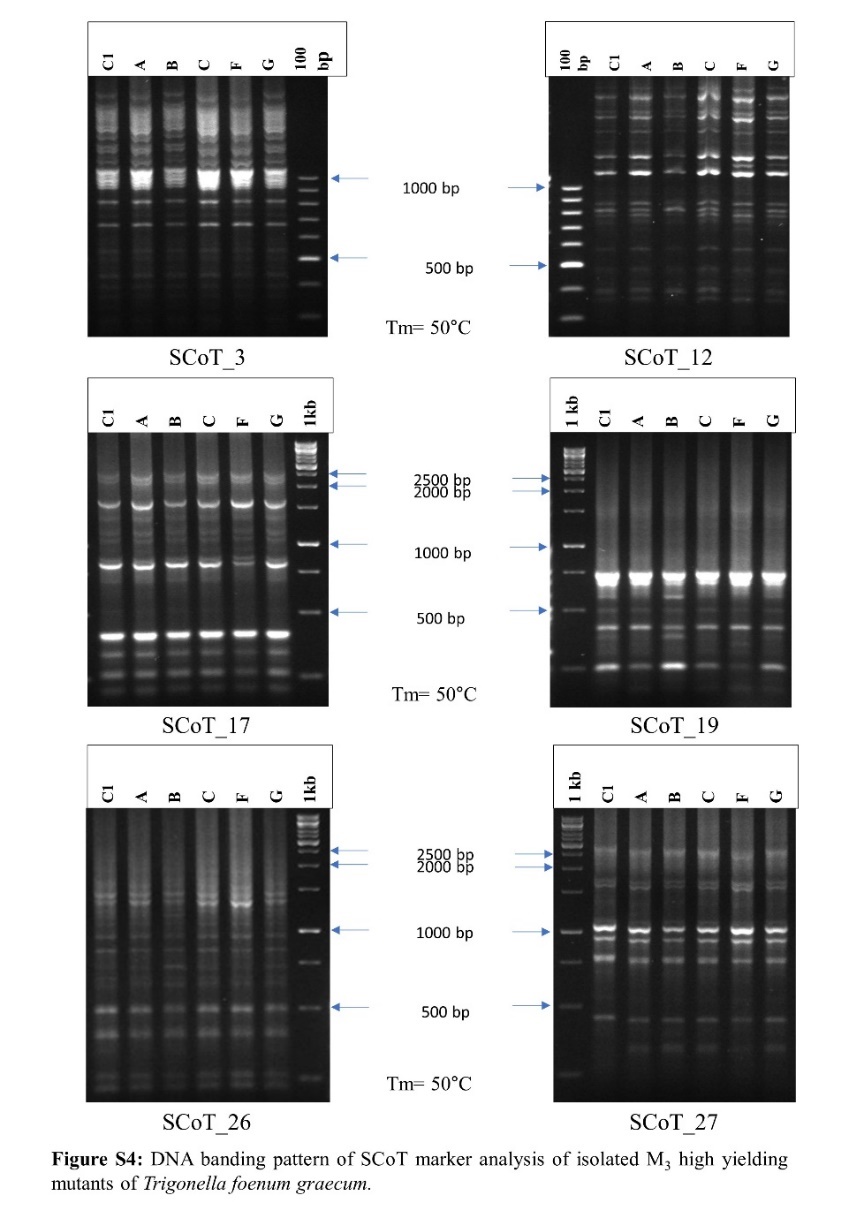


**S2 Fig.: DNA banding pattern of SCoT marker analysis of isolated M_3_ high yielding mutants of *T. corniculata.***

**S1 Fig.: DNA banding pattern of SCoT marker analysis of isolated M_3_ high yielding mutants of *T. foenum-graecum.***

**S4 Table: S**imilarity matrix of high yielding M_3_ mutants of *T. foenum-graecum.*​

|  | C1 | A | B | C | F | G |
| --- | --- | --- | --- | --- | --- | --- |
| C1 | 1.000 |  |  |  |  |  |
| A | 0.875 | 1.000 |  |  |  |  |
| B | 0.889 | 0.816 | 1.000 |  |  |  |
| C | 0.854 | **0.979** | 0.833 | 1.000 |  |  |
| F | 0.796 | 0.917 | **0.740** | 0.896 | 1.000 |  |
| G | 0.872 | 0.917 | 0.851 | 0.936 | 0.875 | 1.000 |

**S5 Table: S**imilarity matrix of high yielding M_3_ mutants of *T. corniculata​*.

|  | C2 | J | L | N | O |
| --- | --- | --- | --- | --- | --- |
| C2 | 1.000 |  |  |  |  |
| J | 0.639 | 1.000 |  |  |  |
| L | **0.581** | 0.818 | 1.000 |  |  |
| N | 0.600 | 0.885 | 0.811 | 1.000 |  |
| O | 0.690 | **0.887** | 0.815 | 0.882 | 1.000 |


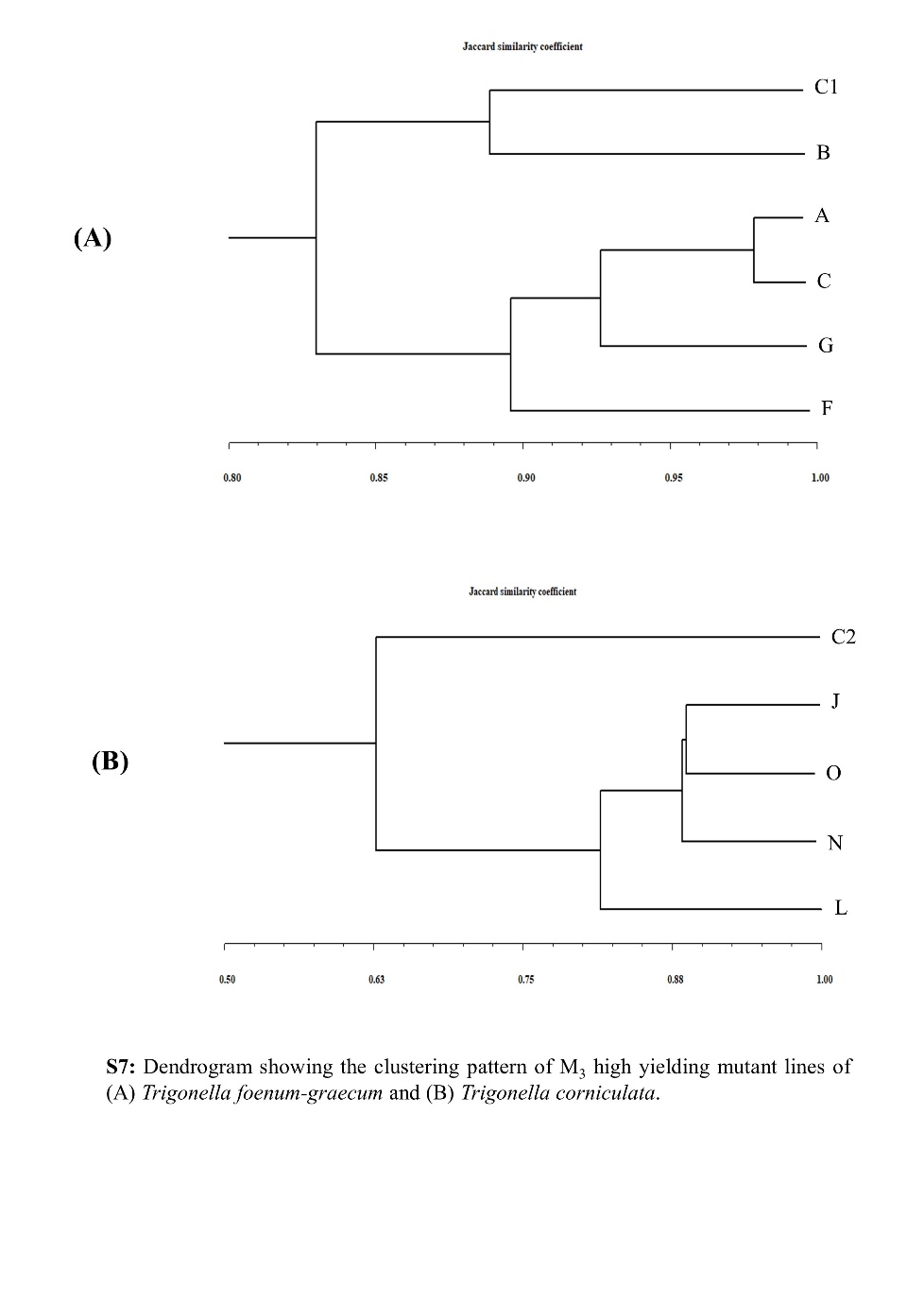


**S3 Fig.**: Dendrogram showing the clustering pattern of M_3_ high yielding mutant lines of (A) *T. foenum-graecum* and (B) *T. corniculata.*
