# Supplementary figures and images for: Biochemical and molecular profiling of induced high yielding M3 mutant lines of two Trigonella species: Insights into improved yield potential
Source: PLoS One. 2024 Jul 29;19(7):e0305691. doi: 10.1371/journal.pone.0305691 (PMC11285971; doi:10.1371/journal.pone.0305691)

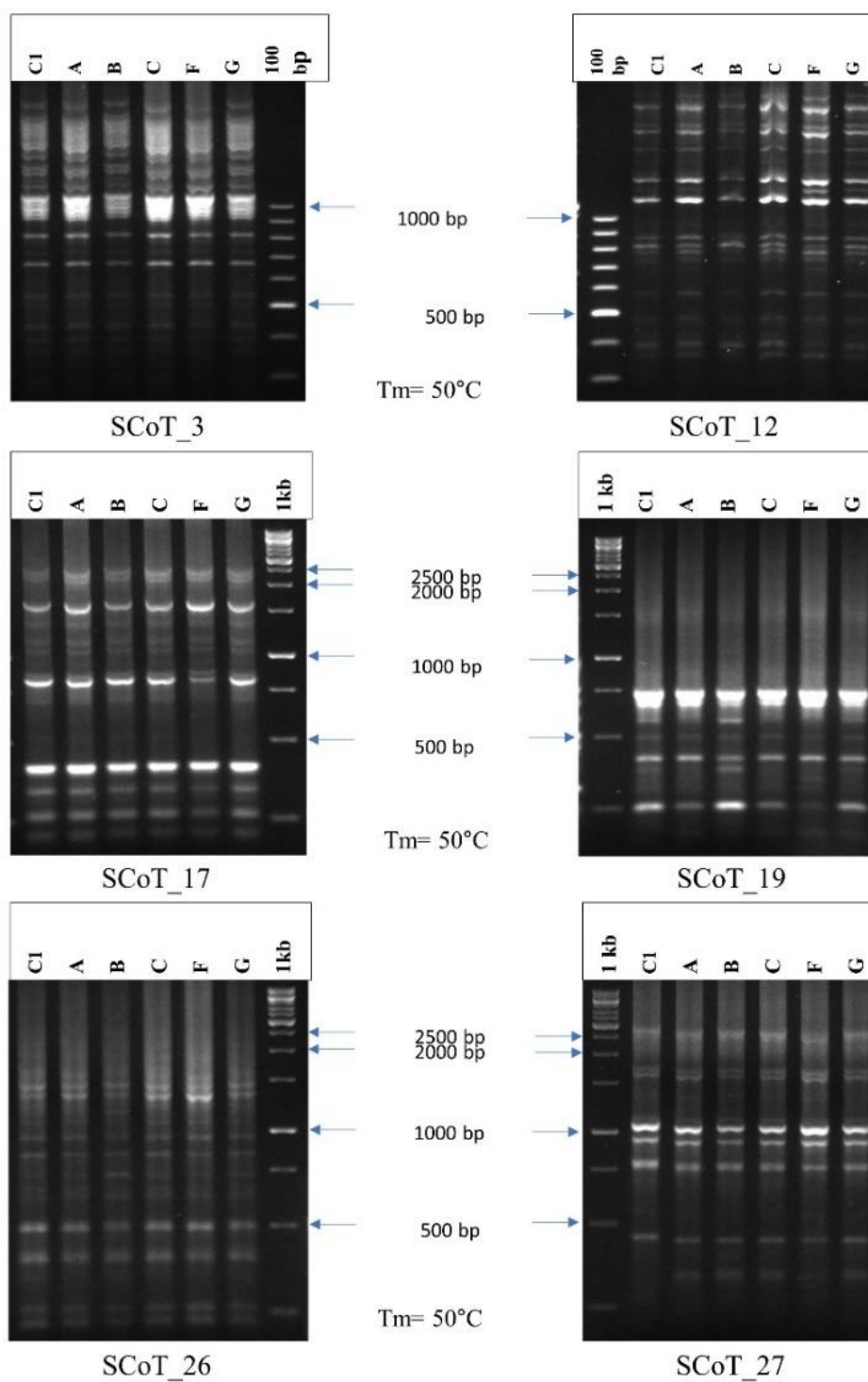

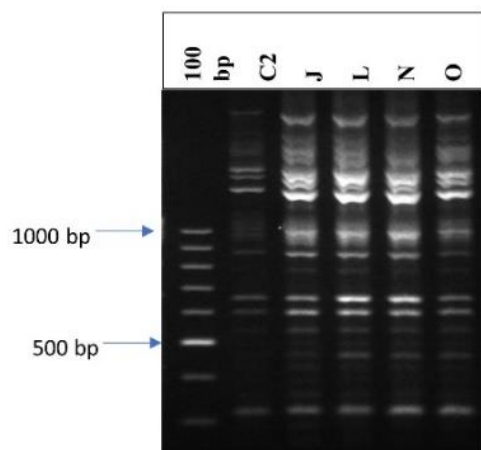

SCoT\_3

$T_m = 50^{\circ}\text{C}$

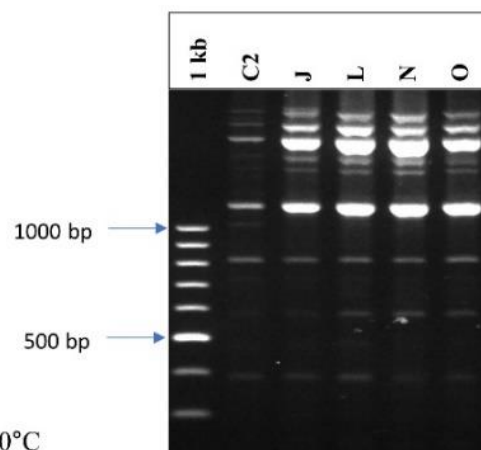

SCoT\_12

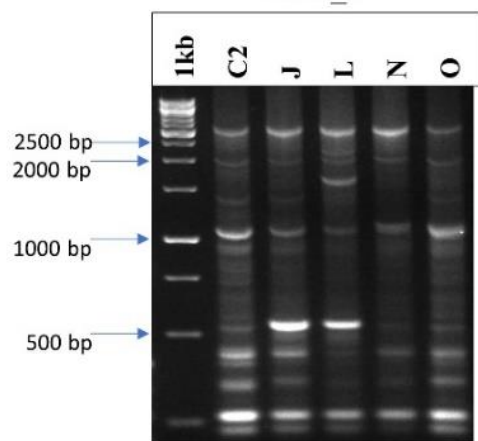

SCoT\_17

$T_m = 50^{\circ}\text{C}$

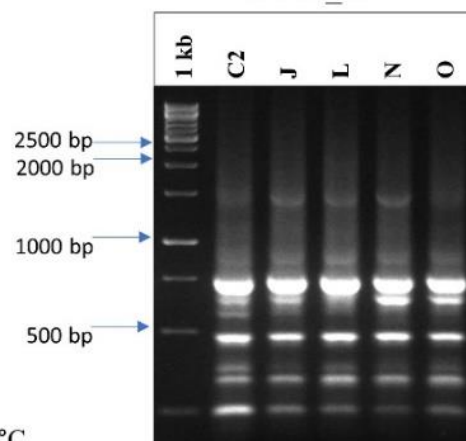

SCoT\_19

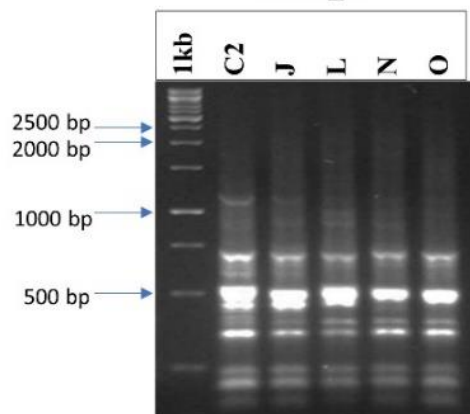

SCoT\_26

$T_m = 50^{\circ}\text{C}$

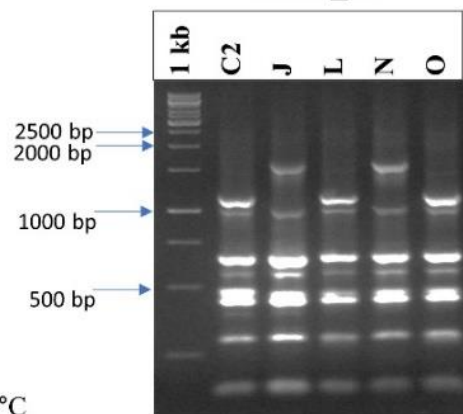

SCoT\_27

Supplement: S1 Raw images — (PDF) [file pone.0305691.s002.pdf]
